# Microbiota Assessments for the Identification and Confirmation of Slit Defect-Causing Bacteria in Milk and Cheddar Cheese

**DOI:** 10.1128/mSystems.01114-20

**Published:** 2021-02-09

**Authors:** Zhengyao Xue, Jason T. Brooks, Zachary Quart, Eric T. Stevens, Mary E. Kable, Jessie Heidenreich, Jeremy McLeod, Maria L. Marco

**Affiliations:** a Department of Food Science and Technology, University of California Davis, Davis, California, USA; b USDA, Agricultural Research Service, Western Human Nutrition Research Center, Immunity and Disease Prevention, Davis, California, USA; c Hilmar Cheese Company, Hilmar, California, USA; University of Naples Federico II

**Keywords:** milk, cheese, microbiota, pasteurization, spoilage, thermoduric bacteria, lactic acid bacteria, heterofermentation, food fermentation, food quality

## Abstract

Validated methods are needed to detect spoilage microbes present in low numbers in foods and ingredients prior to defect onset. We applied propidium monoazide combined with 16S rRNA gene sequencing, qPCR, isolate identification, and pilot-scale cheese making to identify the microorganisms that cause slit defects in industrially produced Cheddar cheese. To investigate milk as the source of spoilage microbes, bacterial composition in milk was measured immediately before and after high-temperature, short-time (HTST) pasteurization over 10-h periods on 10 days and in the resulting cheese blocks. Besides HTST pasteurization-induced changes to milk microbiota composition, a significant increase in numbers of viable bacteria was observed over the 10-h run times of the pasteurizer, including 68-fold-higher numbers of the genus *Thermus*. However, *Thermus* was not associated with slit development. Milk used to make cheese which developed slits instead contained a lower number of total bacteria, higher alpha diversity, and higher proportions of *Lactobacillus*, *Bacillus*, *Brevibacillus*, and *Clostridium*. Only *Lactobacillus* proportions were significantly increased during cheese aging, and *Limosilactobacillus* (*Lactobacillus*) *fermentum*, in particular, was enriched in slit-containing cheeses and the pre- and post-HTST-pasteurization milk used to make them. Pilot-scale cheeses developed slits when inoculated with strains of L. fermentum, other heterofermentative lactic acid bacteria, or uncultured bacterial consortia from slit-associated pasteurized milk, thereby confirming that low-abundance taxa in milk can negatively affect cheese quality. The likelihood that certain microorganisms in milk cause slit defects can be predicted based on comparisons of the bacteria present in the milk used for cheese manufacture.

**IMPORTANCE** Food production involves numerous control points for microorganisms to ensure quality and safety. These control points (e.g., pasteurization) are difficult to develop for fermented foods wherein some microbial contaminants are also expected to provide positive contributions to the final product and spoilage microbes may constitute only a small proportion of all microorganisms present. We showed that microbial composition assessments with 16S rRNA marker gene DNA sequencing are sufficiently robust to detect very-low-abundance bacterial taxa responsible for a major but sporadic Cheddar cheese spoilage defect. Bacterial composition in the (pasteurized) milk and cheese was associated with slit defect development. The application of Koch’s postulates showed that individual bacterial isolates as well as uncultured bacterial consortia were sufficient to cause slits, even when present in very low numbers. This approach may be useful for detection and control of low-abundance spoilage microorganisms present in other foods.

## Abstract (precis)

Food production involves numerous control points for microorganisms to ensure quality and safety. These control points (e.g., pasteurization) are difficult to develop for fermented foods wherein some microbial contaminants are also expected to provide positive contributions to the final product and spoilage microbes may constitute only a small proportion of all microorganisms present.

## INTRODUCTION

Unpasteurized milk contains a large diversity of bacteria originating from animal, human, and environmental sources (1–3). The abundance and proportions of those microorganisms change depending on conditions at the dairy farm and contacts with storage, transport, and processing equipment (4–6). Ultimately, the bacteria in milk, and primarily those that survive pasteurization, impact the quality and safety of the resulting dairy products (7–9).

Cheese is a fermented dairy food that is vulnerable to variations in milk microbiota composition. Although certain bacteria are necessary for cheese manufacture, cheese is also highly susceptible to unwanted bacterial growth and enzymatic activity. Defects (e.g., discoloration, bitterness, and open texture) may occur even when those microbes are not highly abundant (8–10). In Cheddar cheese, slits are one of the most common problems and are defined by open-texture cracks. It is estimated that slit defects cause 180 million dollars in losses each year in the United States alone (11, 12). Slit development is currently unpredictable and happens even when high-quality, low-microbial-count milk is used. The presence of cracks can lead to consumer rejection and additional labor, equipment maintenance, and packaging costs when the cheese is sliced or shredded.

Prior studies examining the causes of Cheddar cheese slit defects have shown that slits are caused by unwanted bacterial growth and excessive gas production by certain nonstarter lactic acid bacteria (LAB) and several *Clostridium* species (13–16). However, the main sources of those spoilage agents were not clearly defined in those studies, potentially due to the use of culture-based enrichment methods, which are now known to be insufficient for identifying the total and spoilage-associated bacterial contents of milk and other foods (17–20).

Culture-independent techniques, such as 16S rRNA gene amplicon DNA sequencing and metagenomics, enable more high-throughput and comprehensive assessments of complex microbial communities. These methods have shown that the bacterial contents of bovine milk vary significantly depending on season and regional climate when the milk was produced as well as on pasteurization and ultrafiltration steps used during processing (4, 17, 21). Nonetheless, nucleic acid-based approaches tend to rely on correlative analysis to infer associations between microbes. Subsequent hypothesis testing with taxa present in complex microbial communities is frequently not possible. Therefore, laboratory culture isolation and DNA sequencing are complementary measures to study milk and cheese microbiota.

We applied culture-dependent and culture-independent methods to investigate the bacterial contents in Cheddar cheese and the raw and pasteurized milk used to make it in order to identify individual isolates and milk-associated bacterial consortia and verify their capacity to cause slits. Because slit development is currently unpredictable, extensive sampling was performed on paired samples of milk (before and after high-temperature, short-time [HTST] pasteurization) and the resulting cheese at a large-scale cheese manufacturer. Milk samples were collected at multiple time points on multiple days, and cheese was tested during aging. Assessments included both the use of propidium monoazide (PMA) in order to detect viable cells (17, 22) and the isolation and identification of thermoduric bacteria and LAB from the milk and cheese. Pilot-scale Cheddar cheese fermentations were used to test the hypothesis that individual bacterial isolates and complete bacterial consortia from slit-associated milk were sufficient to cause slits.

## RESULTS

### HTST pasteurization alters the proportions of viable bacteria in milk.

**FIG 1:**
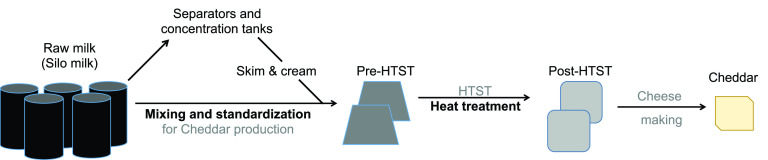
Diagram of the sampling plan. Milk was sampled from storage silos and at the terminal HTST pasteurization step. The microbial shifts that occur during milk mixing and standardization in separators and concentration tanks were documented in a previous study (17). Milk streams entering (pre-HTST) and leaving (post-HTST) the pasteurizer were sampled multiple times throughout 10-h Cheddar production periods and on multiple dates. Cheddar cheese blocks were made using the post-HTST-pasteurization milk and sampled at 0, 30, 90, and 120 days of aging. Slits in cheese were visually examined after aging at 4°C.

**FIG 2:**
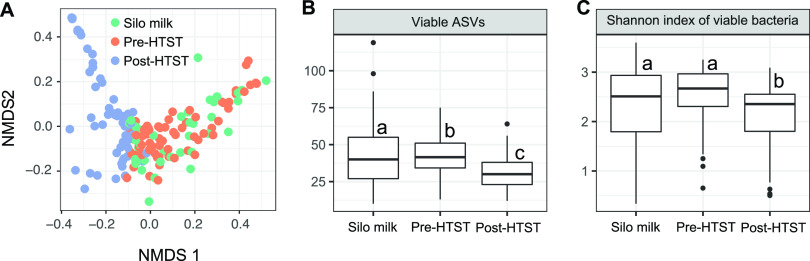
Pasteurization alters the diversity of viable bacteria in milk. (A) Nonmetric multidimensional scaling analysis of individual milk samples. (B and C) Numbers of total observed ASVs (B) and Shannon diversity of each milk type (C) after PMA treatment. Significant differences (Kruskal-Wallis with Dunn test; *P* < 0.05) are indicated by the presence of different lowercase letters above the box plots.

**FIG 3:**
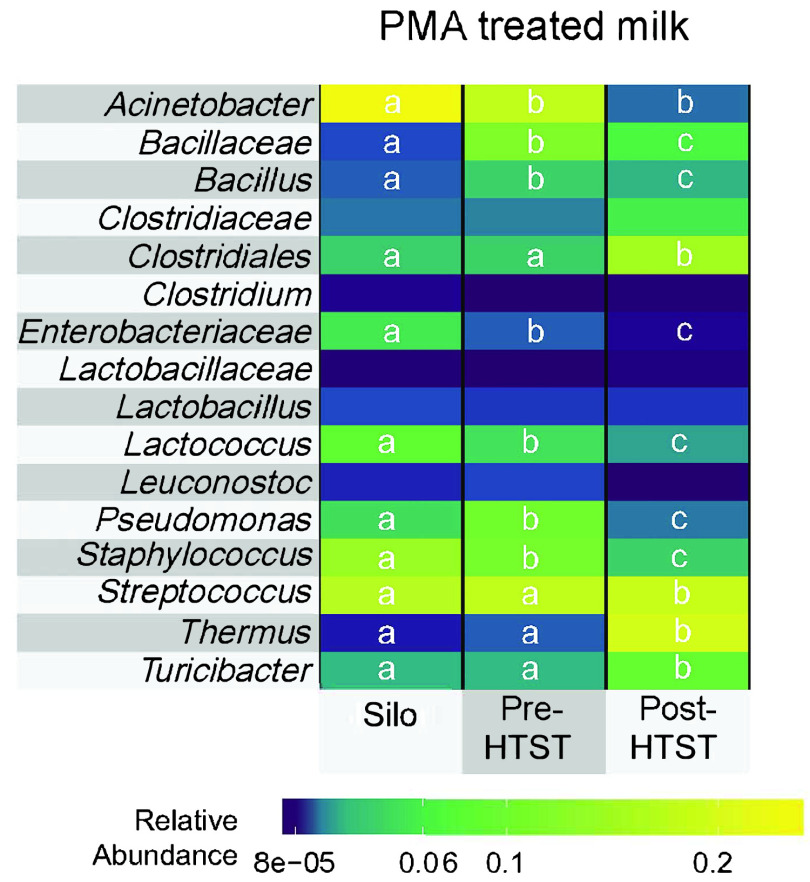
Pasteurized milk is enriched in thermoduric and endospore-forming bacteria. The 16 most abundant bacterial taxa identified from PMA treated samples are shown. The heat map projects the average proportions for 40 samples from silos, 67 samples from pre-HTST pasteurization, and 67 samples from post-HTST pasteurization. Significant differences in the proportions of taxa between collection points (silo, pre-HTST pasteurization, and post-HTST pasteurization) are indicated by lowercase letters (DESeq2 adjusted *P* < 0.1 and log_2_ fold change > 1.25).

The bacteria in milk contained in storage silos and transported into (pre-HTST pasteurization) and out of (post-HTST pasteurization) the HTST pasteurizer immediately prior to Cheddar cheese manufacture were measured on 10 separate days distributed over 4 months (Fig. 1). Besides determining the composition of the total bacterial contents of the milk, a fraction of each milk sample was used to identify viable cells with PMA, a DNA-intercalating dye that hinders PCR amplification from bacteria with a damaged cell membrane (23). Even though milk collected pre-HTST pasteurization had undergone blending, pasteurization, and filtration steps known to cause significant changes in bacterial composition (17), the diversity of viable bacteria in milk pre-HTST pasteurization was more similar to that in the raw, unprocessed milk in storage silos than in milk collected moments later post-HTST pasteurization (Fig. 2A to C). Milk collected from storage silos and pre-HTST pasteurization was enriched in *Staphylococcus*, *Pseudomonas*, *Lactococcus*, *Enterobacteriaceae*, and *Bacillaceae* (Fig. 3). As expected, HTST pasteurization altered the milk microbiota composition (Fig. 2; also, see Fig. S1 in the supplemental material). Milk collected after pasteurization contained significantly higher proportions of viable *Clostridiales* (average of 4.3-fold), *Streptococcus* (average of 1.3-fold), *Thermus* (average of 29.7-fold), and *Turicibacter* (average of 4.3-fold) than milk pre-HTST pasteurization (Fig. 3). Notably, the differences in the silo, pre-HTST pasteurization, and post-HTST-pasteurization milk microbiota were limited to the viable (PMA-treated) cell fractions. There were only slight changes in bacterial composition and no significant differences in alpha diversity when all bacteria (no PMA treatment) from the three collection points were compared (Fig. S1).

10.1128/mSystems.01114-20.2 FIG S1 Alpha diversity of the total bacteria in milk collected at different processing steps. (A) Number of total observed ASVs and (B) Shannon diversity of each milk type. Download FIG S1, PDF file, 0.6 MB. Copyright © 2021 Xue et al. 2021 Xue et al. This content is distributed under the terms of the Creative Commons Attribution 4.0 International license.

### *Thermus* cell numbers increase in milk post-HTST pasteurization over pasteurizer run time.

**FIG 4:**
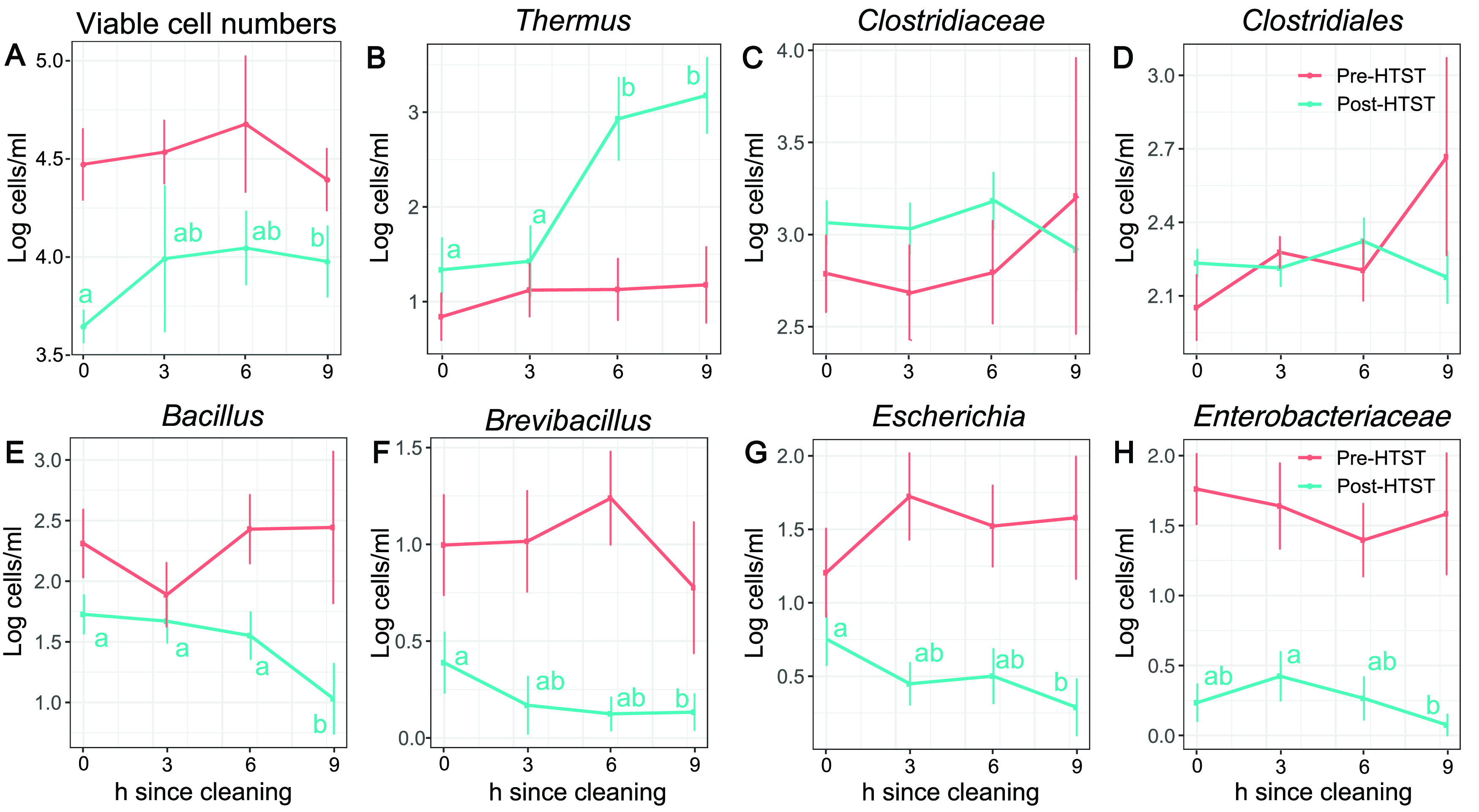
*Thermus* populations increase over time after pasteurizer cleaning. (A) Total bacterial cell numbers (log_10_ transformed) were determined by qPCR on DNA isolated from milk following PMA treatment. (B to H) Cell quantities were estimated by relating the numbers of total viable bacterial cells in a milk sample (A) to the proportions of individual bacterial taxa determined by 16S rRNA gene sequencing. The taxa shown were selected based on their total abundance in milk and variation in cell numbers over time. Lowercase letters indicate significant differences between pre- and post-HTST-pasteurization milk samples according to the Kruskal-Wallis with Dunn test (*P* < 0.05).

We next assessed the composition of the milk microbiota pre- and post-HTST pasteurization in 10-h time courses starting immediately after clean-in-place (CIP) procedures for the pasteurizer. On each of the 10 production days tested, the numbers of viable bacteria in milk post-HTST pasteurization increased from an average of 4.6 × 10^3^ cells/ml after pasteurizer cleaning to 1.3 × 10^4^ cells/ml (*P* < 0.05) 6 h later (Fig. 4A). The increase in cell numbers post-HTST pasteurization was not due to larger amounts of bacteria in milk entering the pasteurizer (pre-HTST pasteurization), because those quantities did not change over time (average of 3.2 × 10^4^ cells/ml) (Fig. 4A). Instead, the higher cell numbers post-HTST pasteurization coincided with a rise in amounts of members of the *Thermus* genus (Fig. 4B). This taxon was present in very low quantities in milk entering the pasteurizer (average of 12 cells/ml). After pasteurization, *Thermus* was present at a level of approximately 20 cells/ml at the start of the production period (time [*t*] = 0 h). This number increased to an average of 850 and 1,500 cells/ml when milk was sampled 6 and 9 h later on all production dates. Conversely, *Clostridiaceae* and other members of the *Clostridiales* were present in similar amounts in milk pre- and post-HTST pasteurization at an average of 250 cells/ml (*Clostridiaceae*) and 720 cells/ml (*Clostridiales*) at all time points (Fig. 4C and D). Additionally, fewer viable *Bacillus*, *Brevibacillus*, and *Enterobacteriaceae* were present in milk post-HTST pasteurization than pre-HTST pasteurization, and their numbers continued to decline during pasteurizer run times on each of the production days (Fig. 4E to H).

### Nonstarter *Lactobacillus* cell numbers increase during Cheddar cheese aging.

**FIG 5:**
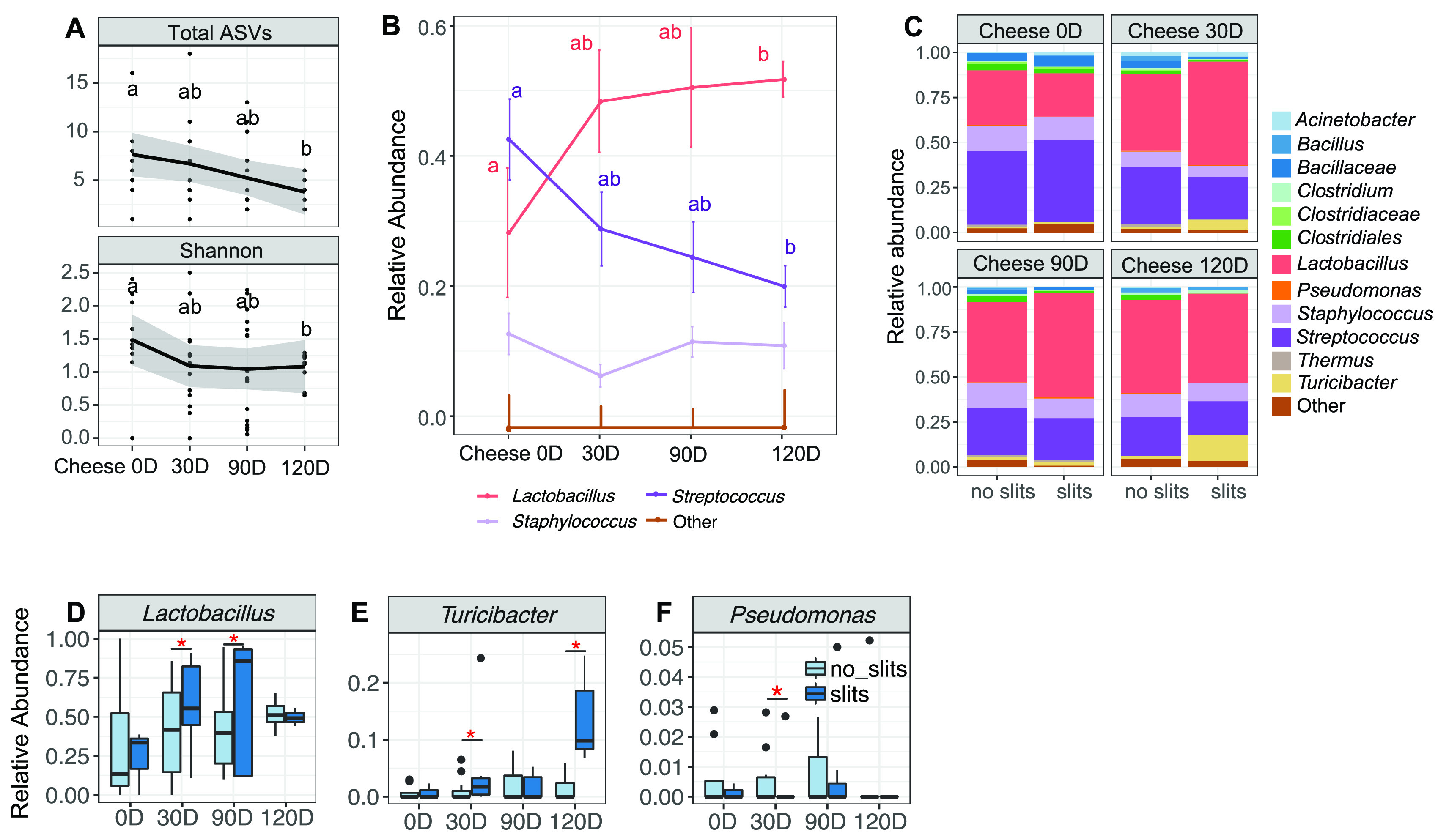
*Lactobacillus* cell numbers increase with cheese aging and are associated with cheese slits. Starter culture *Lactococcus* DNA sequences were removed prior to analysis. (A) Alpha diversity plotted as observed total ASVs and Shannon index; (B and C) proportions of the viable bacteria in Cheddar cheese; (D to F) proportions of viable *Lactobacillus*, *Turicibacter*, and *Pseudomonas*, respectively. These were the only taxa found in significantly different proportions between cheese with and without slits. Bacteria in PMA-treated cheese samples are shown. In panels B and C, the most abundant bacterial taxa are named and all the other bacteria are grouped in the “other” category. Significant differences are indicated by different lowercase letters or asterisks and determined by (A) the Kruskal-Wallis with Dunn test (*P* < 0.05) and (B to F) a DESeq2 adjusted *P* value of <0.1 and a log_2_ fold change of >1.25.

The microbial contents of Cheddar cheese made from milk that was pasteurized 3 h after HTST pasteurizer cleaning was monitored for 120 days (Fig. 5A). As expected, Lactococcus lactis, the organism used as the cheese starter culture, was very abundant and constituted, on average, 96% of the viable bacteria present (89.91% to 99.78%) in the aged cheese according to DNA sequence analysis. The nonstarter, viable bacterial contaminants in the cheese constituted the remaining 0.22% to 10.09% of all DNA sequence reads. The nonstarter bacteria were predominantly *Lactobacillus* (44.3% ± 31.1%), *Streptococcus* (29.2% ± 15.8%), and *Staphylococcus* (10.4% ± 9.8%) (Fig. 5B). Only the proportions of *Lactobacillus* increased over the 120 days of aging (Fig. 5B). The levels of *Streptococcus* declined over time, whereas *Staphylococcus* populations remained constant.

### *Lactobacillus* and *Turicibacter* are associated with Cheddar cheese slit defects.

Within 90 days of aging, four of the 10 cheese blocks developed slits. All cheese blocks were similar according to the general quality characteristics of pH (average pH of 5.12), fat content (33.2%), moisture (38.1%), lactose (0.19%), and l-lactate (1.01%) (Fig. S2). An exception to this finding was that cheeses which developed slits contained modest but significant reductions in salt content at 5 days (normal cheese, 1.81% ± 0.09%; cheese with slits, 1.74% ± 0.09%; *P* = 0.015) and 90 days (normal cheese, 1.79% ± 0.07%; cheese with slits, 1.70% ± 0.09%; *P* = 0.017) of aging (Fig. S2).

10.1128/mSystems.01114-20.3 FIG S2 Chemical analysis of Cheddar cheese at (A) 5 days and (B) 90 days. All measurements except pH are cheese percentages (per weight). Citrate concentration was not collected for 5-day-old cheese with slits. Significant differences were determined according to the *t* test (*P* < 0.05). Download FIG S2, PDF file, 0.1 MB. Copyright © 2021 Xue et al. 2021 Xue et al. This content is distributed under the terms of the Creative Commons Attribution 4.0 International license.

Cheeses that developed slits contained an altered nonstarter microbiota (Fig. 5C). Whereas good-quality cheese harbored significantly higher proportions of viable *Pseudomonas* after 30 days of aging (Fig. 5F), cheeses with slits were found to have higher proportions of *Lactobacillus* (average of 1.4-fold) and *Turicibacter* (average of 4.4-fold) at that time point (Fig. 5D and E). *Lactobacillus* and *Turicibacter* were also enriched at 90 and 120 days of aging, respectively (Fig. 5D and E). The trend toward higher numbers of *Turicibacter* spp. in cheese with slits (averages of 1.2- and 2.2-fold at 90 and 120 days, respectively) was confirmed by qPCR (Fig. S3A). The same was found for *Limosilactobacillus* (*Lactobacillus*) *fermentum* (average of 1.1-fold at 120 days), the species identified as the dominant *Lactobacillus* amplicon sequence variant (ASV) in the cheese (Fig. S3B).

10.1128/mSystems.01114-20.4 FIG S3 Taxa-specific qPCR results of pre- and post-HTST-pasteurization milk, and cheese. qPCR assays were designed with specific primers targeting *Turicibacter* spp. (A) and *L. fermentum* in aging cheese (B) and pre- and post-HTST-pasteurization milk (C). Results from PMA-treated samples are shown. Significant differences were determined according to the Mann-Whitney test (*P* < 0.05). Download FIG S3, PDF file, 0.2 MB. Copyright © 2021 Xue et al. 2021 Xue et al. This content is distributed under the terms of the Creative Commons Attribution 4.0 International license.

### *Lactobacillus* in milk pre- and post-HTST pasteurization corresponds to Cheddar cheese slit defects.

**FIG 6:**
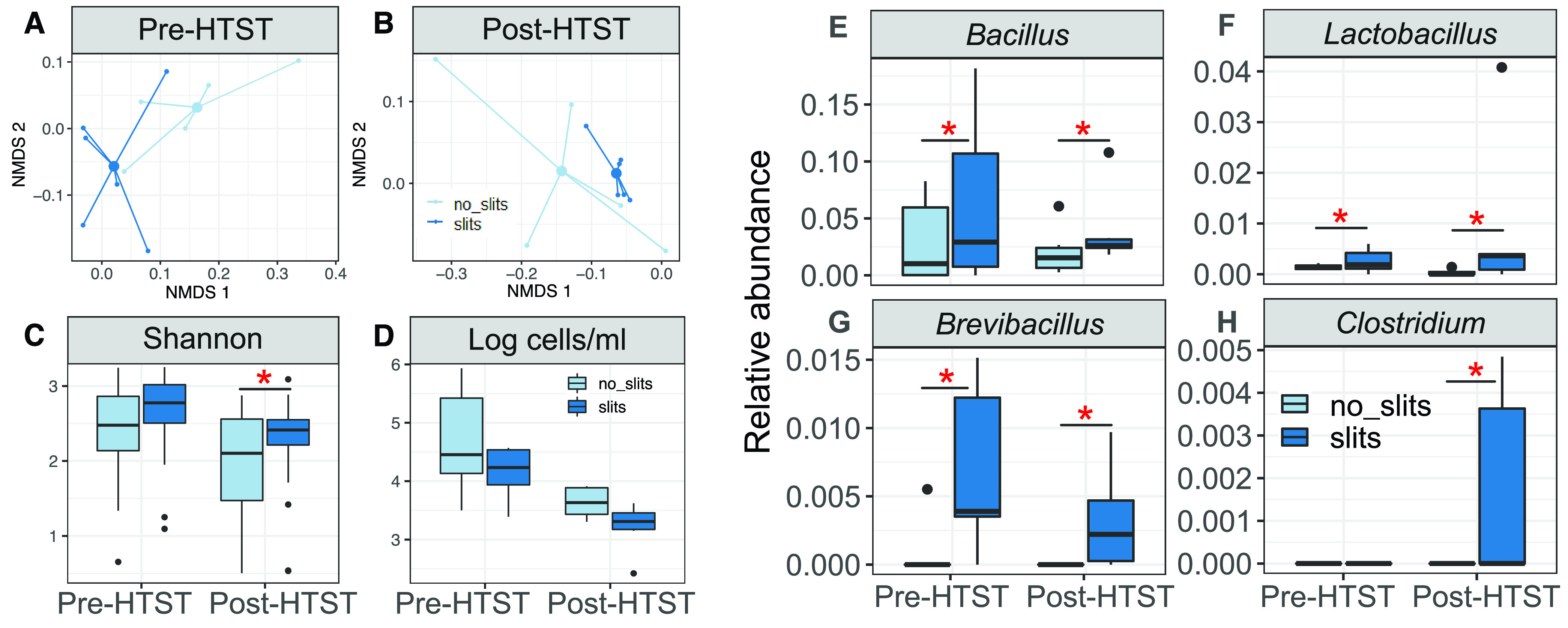
Pre-HTST and post-HTST-pasteurization milk microbiotas are associated with Cheddar cheese slit defects. (A and B) Nonmetric multidimensional scaling analysis; (C) Shannon diversity; (D) log_10_-transformed numbers of viable bacteria; (E to H) proportions of viable *Bacillus*, *Lactobacillus*, *Brevibacillus*, and *Clostridium*, respectively. These were the only taxa found to be significantly enriched in milk associated with cheese slits. Asterisks indicate significant differences between milk samples according to (C and D) the Mann-Whitney test (*P* < 0.05) and (E to H) a DESeq2 adjusted *P* value of <0.1 and a log_2_ fold change of >1.25.

The pre- and post-HTST-pasteurization milk used to make the cheese blocks that developed slits contained a different microbial composition than milk that resulted in good-quality cheese (Fig. 6A and B). These differences accounted for most of the variation observed between milk samples (pre-HTST-pasteurization milk, Adonis *R*^2^ = 0.29, *P* = 0.018; post-HTST-pasteurization milk, Adonis *R*^2^ = 0.21, *P* = 0.046). The Shannon index of the viable bacteria was significantly higher in post-HTST-pasteurization milk associated with slits in cheese (Fig. 6C), and those milk samples tended to have lower cell numbers (*P* = 0.087) (Fig. 6D). The proportions of several bacterial taxa also differed (Fig. S4A), and as found for the cheeses, *Pseudomonas* proportions were higher (average of 2.0-fold) in pre-HTST-pasteurization milk that resulted in good-quality cheese (Fig. S4A). Proportions of Acinetobacter were also similarly elevated (average of 5.2-fold) post-HTST-pasteurization milk (Fig. S4B). *Lactobacillus*, *Brevibacillus*, and *Bacillus* were significantly enriched in pre-HTST-pasteurization milk (averages of 1.8-, 14.7-, and 2.1-fold, respectively) and post-HTST-pasteurization milk (averages of 26.1-, 30.0-, and 1.9-fold, respectively) associated with cheese slits (Fig. 6E to G). *Clostridium* was also similarly enriched but only in milk samples collected post-HTST pasteurization (Fig. 6H). Unlike what was found for the cheese, *Turicibacter* was not enriched in milk that was used to make cheese that developed slits.

10.1128/mSystems.01114-20.5 FIG S4 Proportions of *Pseudomonas* and Acinetobacter in pre- and post-HTST-pasteurization milk resulting in normal and slit-containing Cheddar cheese. Proportions of (A) *Pseudomonas* and (B) Acinetobacter in PMA-treated cheese samples are shown. Asterisks indicate significant differences between milk samples according to a DESeq2 adjusted *P* value of <0.1 and a log_2_ fold change of >1.25. Download FIG S4, PDF file, 0.2 MB. Copyright © 2021 Xue et al. 2021 Xue et al. This content is distributed under the terms of the Creative Commons Attribution 4.0 International license.

*Lactobacillus* was the only member of the milk microbiota that was consistently enriched in both slit-containing cheese (Fig. 5D) and the pre- and post-HTST-pasteurization milk used to make it (Fig. 6F). Odds ratio analysis also supported this result (Fig. S5). At the species level, *L. fermentum* was the most abundant *Lactobacillus* ASV in milk both pre- and post-HTST pasteurization. This species was enriched in milk resulting in slit-containing cheese blocks (Fig. S3C) with an effect size of 0.92 {effect size = [(mean_no_slits_ − mean_slits_)/standard deviation]}. Assuming that effect size remains constant, approximately 100 milk samples should be collected to estimate the numbers of *L. fermentum* in order to predict slit formation in cheese at an accuracy of 95% (Fig. S6).

10.1128/mSystems.01114-20.6 FIG S5 Biomarker identification according to odds ratio analysis of the bacterial contents in cheese and pre- and post-HTST-pasteurization milk. Bacteria in PMA-treated milk and cheese samples are shown. Each whisker plot represents the mean of the log_10_(odds ratio) and 2.5% to 97.5% quantile. Download FIG S5, PDF file, 0.4 MB. Copyright © 2021 Xue et al. 2021 Xue et al. This content is distributed under the terms of the Creative Commons Attribution 4.0 International license.

10.1128/mSystems.01114-20.7 FIG S6 Power as a function of total number of samples. The significance level was set to 0.05 for a single-tailed comparison (“greater”) between the two groups, with the assumption that the slit containing cheese harbors higher numbers of *Limosilactobacillus fermentum*. Total number of samples = sample size × 2. Download FIG S6, PDF file, 0.1 MB. Copyright © 2021 Xue et al. 2021 Xue et al. This content is distributed under the terms of the Creative Commons Attribution 4.0 International license.

### Diverse LAB and thermoduric bacteria are present in pasteurized milk and cheese.

Milk collected pre- and post-HTST pasteurization on several dates in 2017 and 2018 and the resulting corresponding Cheddar cheese blocks were used to isolate culturable bacteria. As found in 2015, several of the cheese blocks tested developed slits (Table S1). Because of the association of LAB and thermoduric bacteria (endospore formers and others) with slits, those two groups were the focus of culture-based enrichments. A total of 69 LAB isolates were collected, including *Lactiplatibacillus* (*Lactobacillus*) *plantarum* (16 isolates), *L. fermentum* (11 isolates), Leuconostoc lactis (6 isolates), and Leuconostoc mesenteroides (6 isolates) (Table S1). Bacteria able to survive at 80°C for 20 min encompassed 207 isolates belonging to 43 different bacterial species (Table S1). Most of the isolates were identified as members of the genus *Bacillus* and included high numbers of representatives of Bacillus licheniformis (41 isolates) and Bacillus paralicheniformis (30 isolates). Besides *Bacillus*, other endospore-forming bacteria were isolated, including *Paenibacillus* sp. and Brevibacillus brevis. Isolates also included Serratia liquefaciens, Pseudomonas fragi, and Pseudomonas psychrophila, species in the phylum *Proteobacteria*. Those taxa were isolated from milk pre-HTST pasteurization and not from milk post-HTST pasteurization or cheese.

10.1128/mSystems.01114-20.1 TABLE S1 Numbers of LAB and thermoduric bacteria isolated from milk before (pre-HTST) and after (post-HTST) pasteurization and from Cheddar cheese. Download Table S1, DOCX file, 0.02 MB. Copyright © 2021 Xue et al. 2021 Xue et al. This content is distributed under the terms of the Creative Commons Attribution 4.0 International license.

Because cheese slit formation is caused by the accumulation of gas (CO_2_), the capacity of the bacterial isolates to produce gas during growth was measured. The heterofermentative LAB, *L. fermentum*, *Ln. mesenteroides*, and *Ln. lactis*, produced gas during growth in MRS broth-containing Durham tubes. Gas production was not visible for isolates of the homofermentative LAB species Lactobacillus delbrueckii subsp. *lactis*, Lactobacillus paracasei, Lactobacillus paraplantarum, L. plantarum, and Lactobacillus rhamnosus as well as thermoduric bacteria. The heterofermentative LAB were then examined for thermotolerance and hence the capacity to survive HTST pasteurization. Whereas all three *L. fermentum* isolates tested survived simulated HTST pasteurization conditions for 15 s at 72°C (Fig. S7), none of the *Ln. mesenteroides* and only one of the three *Ln. lactis* isolates survived for that length of time (Fig. S7).

10.1128/mSystems.01114-20.8 FIG S7 Thermal tolerance of lactic acid bacterial isolates. Heat treatment was performed at 72°C. Each data point represents the mean and standard deviation from three replicate cultures measured prior to exposure (*t* = 0) and after 15 and 60 s at 72°C. The dashed horizontal line denotes the detection limit (60 CFU/ml). Download FIG S7, PDF file, 0.2 MB. Copyright © 2021 Xue et al. 2021 Xue et al. This content is distributed under the terms of the Creative Commons Attribution 4.0 International license.

### Heterofermentative LAB and milk consortia cause slit formation in Cheddar cheese.

*L. fermentum*, *Ln. mesenteroides*, and *Ln. lactis* isolates (3 isolates of each species) were tested for their role in the development of Cheddar cheese slit defects. Rifampin-resistant variants of each of the isolates along with *L. plantarum* NCIMB8826-R were inoculated at either moderate (10^7^ CFU/g) or high (10^9^ CFU/g) levels into freshly made cheese curds prior to pressing and aging. The lower cell quantities were in the range of *Lactobacillus* cell numbers found in Cheddar cheese (2.5% ± 1.2% of the 10^9^ CFU/g viable bacteria in the 2015 cheese samples). After aging, culturable bacterial cell numbers decreased from 10^9^ CFU/g to 10^8^ CFU/g (Fig. S8). The quantities of the rifampin-resistant LAB inoculants remained unchanged in cheese inoculated with 10^7^ CFU/g and decreased by 10-fold in cheese into which 10^9^ CFU/g were added (Fig. S8).

10.1128/mSystems.01114-20.9 FIG S8 Bacterial cell counts in inoculated pilot cheese. Cheddar cheeses were inoculated with (A) 10^7^ CFU/g or (B) 10^9^ CFU/g of individual bacterial strains or (C) cryopreserved bacterial consortia from milk or saline (controls) in triplicate. Grey tabs above the top panel indicate the inocula, and grey tabs on the right indicate the bacterial group of interest. Significant differences were determined according to the Kruskal-Wallis with Dunn test (*P* < 0.05). Download FIG S8, PDF file, 0.1 MB. Copyright © 2021 Xue et al. 2021 Xue et al. This content is distributed under the terms of the Creative Commons Attribution 4.0 International license.

**FIG 7:**
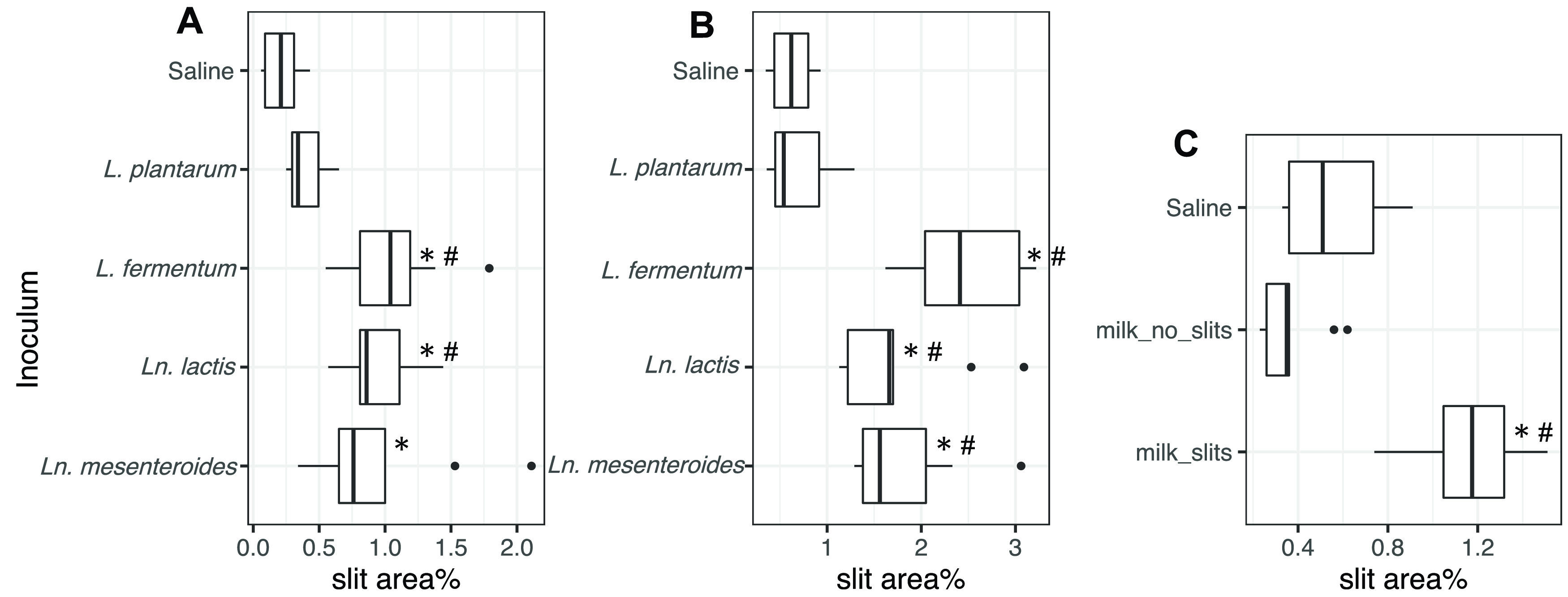
Inoculation of heterofermentative LAB or slit-associated milk consortia results in Cheddar cheese with slits. The percentage of surface area containing slits is shown for cheese inoculated with either (A) 10^7^ CFU/g or (B) 10^9^ CFU/g of the indicated LAB species or with (C) cryopreserved milk bacterial consortia. For each cheese sample, three cross-section surfaces were randomly selected for slit analysis. The Kruskal-Wallis with Dunn test (*P* < 0.05) was used to assess for significant differences in slit area compared to controls with added saline (*) (A, B, and C), the homofermentative LAB *L. plantarum* (#) (A and B), or bacterial consortia from milk not associated with cheese slit formation (*) (C).

**FIG 8:**
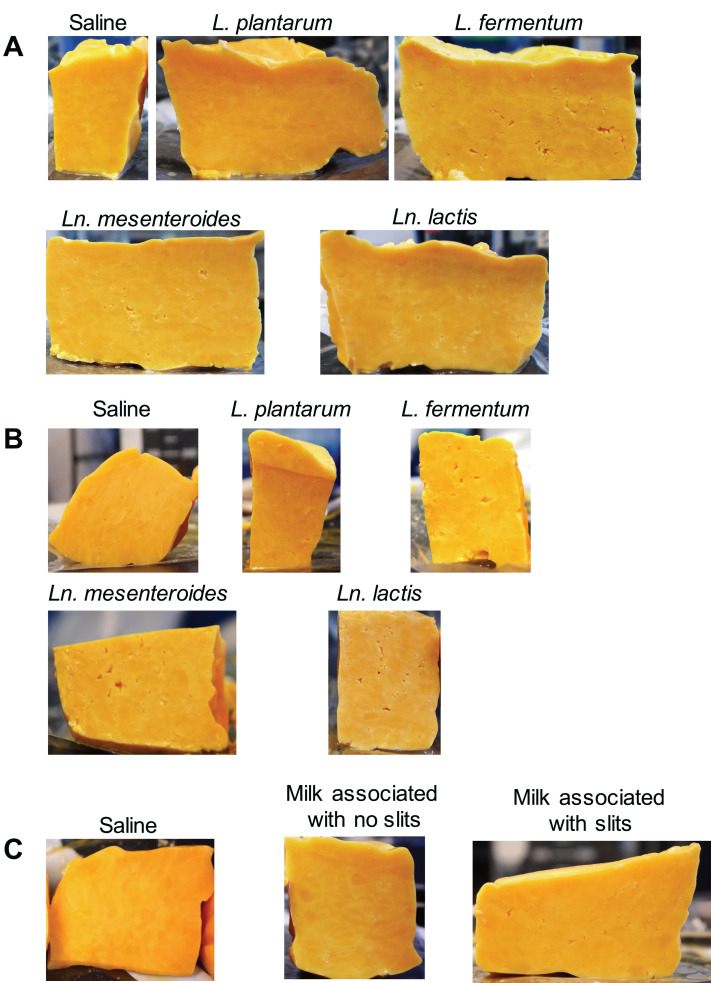
Representative photos of pilot cheese cross sections. Cheese was inoculated with (A) 10^7^ CFU/g or (B) 10^9^ CFU/g of the selected bacterial strains or (C) with cryopreserved milk consortia. Saline was added to serve as an inoculum control.

Slits were found in all cheese blocks inoculated with either *L. fermentum*, *Ln. mesenteroides*, or *Ln. lactis* (Fig. 7A and B and 8A and B). Although only a small fraction of the cheese surface area developed slits, this level of damage was visible and comparable to slit quantities regarded to be significant quality defects in commercial Cheddar cheese, which are approximately 85 cm^2^ and 520 cm^2^ in 40-lb and 700-lb cheese, respectively (data not shown). Cheese containing the *L. fermentum* isolates exhibited the highest levels of slit damage. The surface area of those cheeses contained 4- to 5-fold-higher levels of slits than the controls (Fig. 7A and B and 8A and B). A dose-dependent effect was also observed such that cheese inoculated with *L. fermentum* at a level of 10^9^ CFU/g contained more slits (2.5% ± 0.6% cheese area) than cheese inoculated with 10^7^ CFU/g (1.1% ± 0.4% cheese area) (Fig. 7A and B). Culture-independent assessments of the cheese microbiota by DNA sequencing showed that *L. fermentum* was an abundant ASV (Fig. S9A to C). Notably, cheese blocks inoculated with the *Ln. mesenteroides* and *Ln. lactis* isolates also contained elevated levels of nonstarter *L. fermentum* (Fig. S9A and B), potentially indicating that those bacteria altered the cheese in a manner that promoted *L. fermentum* growth.

10.1128/mSystems.01114-20.10 FIG S9 Proportions of *L. fermentum*, *Lactobacillaceae*, and *Leuconostoc* in pilot cheese. Cheddar cheeses were inoculated with (A) 10^7^ CFU/g or (B) 10^9^ CFU/g of individual bacterial strains or (C) cryopreserved bacterial consortia from milk or saline (controls) in triplicate. Significant differences are represented by different lowercase letters. Statistical significance was determined according to a DESeq2 adjusted *P* value of <0.1 and a log_2_ fold change of >1.25. PMA-treated cheese samples were used for bacterial identification. Lnl, *Ln. lactis*; Lnm, *Ln. mesenteroides*; Lf, *L. fermentum*; Lp, *L. plantarum*. Download FIG S9, PDF file, 0.3 MB. Copyright © 2021 Xue et al. 2021 Xue et al. This content is distributed under the terms of the Creative Commons Attribution 4.0 International license.

We also tested whether the inoculation of cryopreserved bacterial consortia collected from milk post-HTST pasteurization and not cultured in laboratory medium could cause slit defects. Consortia were selected because they were either associated with good-quality cheese (no slits) or cheese with slit defects. Adding those bacteria to cheese curds and then pressing and aging confirmed that slit-associated milk consortia are sufficient to cause slits (Fig. 7C and 8C). Importantly, because the inocula consisted of only approximately 1.8 × 10^4^ cells, this finding showed that slit production was reproducible and only low numbers of contaminating bacteria are needed to cause this defect. DNA sequencing confirmed that the proportions of *L. fermentum* ASVs were higher in cheese made using slit-associated milk consortia than in cheese inoculated with bacteria from milk that yielded good-quality cheese (Fig. S9C).

## DISCUSSION

Food quality defects are significant contributors to the global problem of food waste. Detecting the cause of microbe-induced fermented-food spoilage is particularly challenging because of the expected large number and frequently diverse microorganisms present. Herein, we show that culture-independent microbiota assessments with viable-cell-targeted 16S rRNA gene amplicon DNA sequencing are useful for the identification of low-abundance taxa responsible for slit defects in Cheddar cheese and that certain thermotolerant bacterial contaminants in milk used to make that cheese are responsible for those slits. These findings demonstrate that although there are significant challenges to the detection of microbial spoilage contaminants in foods and food ingredients, microbiota assessments with 16S rRNA marker gene sequencing are sensitive and have both predictive and diagnostic value.

Focusing on the final HTST pasteurizer step prior to cheese manufacture, we found that HTST pasteurization results in reductions in viable-cell numbers and bacterial diversity as well as changes to the proportions of individual bacterial taxa. Notably, our capacity to identify these differences was specific to the PMA-treated milk, which resulted in selective detection of viable bacterial populations. This finding is consistent with our prior observation that pasteurization does not modify the total bacterial DNA contents of milk but rather alters the proportions of the remaining surviving cells (17). Also as reported previously, pasteurization yielded lower proportions of *Staphylococcus* and *Pseudomonas* and higher levels of endospore-forming bacteria, including *Turicibacter* and *Clostridiales* (17).

Viable-cell numbers and quantities of individual bacterial taxa varied in milk post-HTST pasteurization over 10-h pasteurizer run times. The changes were remarkably consistent considering that the sampling time course was performed on different days spanning over 4 months. Because the bacterial composition in pre-HTST-pasteurization milk remained relatively constant, it is likely that the differences post-HTST pasteurization were the result of retention, growth, and/or release of bacteria from the pasteurizer. While quantities of clostridia in milk post-HTST pasteurization did not change over time, levels of other endospore-forming taxa such as *Bacillus* declined. On the other hand, numbers of bacteria in the genus *Thermus* increased. *Thermus*, a member of the phylum *Deinococcus-Thermus*, was previously found in milk (24, 25), and at least one species, Thermus thermophilus, is a thermophile that can grow between 47°C and 85°C with an optimum growth range between 65°C and 72°C (26). Because the HTST pasteurizer temperature was within that range, it is possible that *Thermus* grew inside the equipment. Previous studies confirmed that the microbial populations on processing equipment surfaces are closely correlated with the microorganisms present in the final food contents (5, 6, 27). Because T. thermophilus can cause a pink discoloring defect (8), pasteurizer testing could be useful for preventing other types of cheese spoilage.

Despite its abundance in post-HTST-pasteurization milk, *Thermus* was not associated with Cheddar cheese slit defects. *Bacillus*, *Brevibacillus*, *Clostridium*, and *Lactobacillus* were instead detected in higher numbers in pre- and/or post-HTST-pasteurization milk associated with cheese slits. *Bacillales*, including members of the genera *Bacillus*, *Brevibacillus*, and *Paenibacillus*, were also isolated from milk and cheese. These bacteria are frequently present in milk, dairy processing plants, and dairy products (28–30). Although not known to cause slit defects, *Bacillales*, including B. licheniformis, Bacillus subtilis, and Bacillus amyloliquefaciens, may produce biogenic amines (31, 32) and cause off flavors due to lipolysis or proteolysis (29, 33, 34). Thus, *Bacillales* remain important contaminants to monitor in milk and dairy foods (28). Among the clostridia, Clostridium tyrobutyricum may cause slits (35, 36). However, because no off flavor or odor defects were detected (16, 35, 37), it is unlikely that *C. tyrobutyricum* and other *Clostridium* species were the primary spoilage agents of the cheese studied here. Conversely, *Lactobacillus*, primarily represented by heterofermentative LAB and specifically *L. fermentum*, were the only members of the milk and cheese microbiota that were positively correlated with slit defects according to 16S rRNA gene sequence analysis. This finding is consistent with prior studies suggesting that *L. fermentum* is associated with slit development in Cheddar cheese (14).

Although *Turicibacter* was also enriched in slit-containing cheese, *Turicibacter* levels in milk were not correlated with slit development, nor was this genus enriched in slit-containing pilot Cheddar cheese. While we cannot rule out the capacity of *Turicibacter* to cause slits, additional work will be needed to identify and isolate members of this fastidious genus (38). *Turicibacter* appears to be pervasive in bovine milk and was present in proportions over 1% in numerous studies investigating the microbiota of bovine milk and dairy products using culture-independent methods (4, 39–42). Additionally, although the salt content of slit-containing cheese was lower than that found in normal cheese blocks, the differences were quite small (<0.1%). Therefore, further study is needed to understand the extent to which small variations in salt content may contribute to slit development.

To test the hypothesis that 16S rRNA gene amplicon DNA sequencing is sufficiently sensitive to identify the low-abundance taxa (2.5% ± 1.2% of bacteria present; <50 cells/ml) responsible for slit defects and that the contaminant bacteria in milk cause them, pilot cheeses were inoculated with isolates of heterofermentative LAB isolated from pre- and post-HTST-pasteurization milk. All three strains of each of the three heterofermentative LAB species examined (*L. fermentum*, *Ln. mesenteroides*, and *Ln. lactis*) resulted in slit damage. Remarkably, those pilot-scale cheeses developed slits even when low numbers of those strains or the uncultured, slit-associated bacterial consortia were inoculated. Because cheese inoculated with the homofermentative LAB *L. plantarum* did not develop slits, the results also indicate that the capacity for that type of cheese defect is a general risk of heterofermentative LAB growth. Unlike homofermentative LAB, which rely on glycolysis for sugar metabolism and energy generation, heterofermentive LAB utilize the phosphoketalase pathway, which results in CO_2_ synthesis as a result of decarboxylation of 6-phosphogluconate. The likelihood that *L. fermentum* was the primary spoilage agent at this particular creamery is supported by the high thermal tolerance levels observed for the strains tested here. *L. fermentum* was also present at higher proportions in pilot cheese inoculated with the cryopreserved bacterial consortia from milk used to make the Cheddar that showed slit defects during aging. Although *Leuconostoc* was below the detection limit in HTST-pasteurized milk by both 16S rRNA gene sequencing and culture-dependent methods, thermotolerant *Leuconostoc* and other heterofermentative LAB should remain targets for measurement and control because of their slit-forming potential.

In conclusion, our investigations of the bacterial contents of pre- and post-HTST-pasteurization milk and Cheddar cheese produced at a large-scale dairy processing facility illustrate the usefulness of 16S rRNA gene amplicon DNA sequencing on viable cell fractions for detecting low-abundance spoilage bacteria present in food ingredients. This approach was useful despite the limitations of marker gene DNA sequencing to identify some bacteria to the species or even genus level. Shotgun metagenomics may provide some additional information on the functional capacities of microorganisms in milk and Cheddar cheese. However, this technique is currently cost-prohibitive and requires high sequencing coverage to detect low-abundance spoilage microorganisms within a diverse contaminant microbiota and genomic DNA from plant or animal sources. In order to prevent slit defects, 16S rRNA gene amplicon DNA sequencing and targeted methods such as qPCR should be incorporated into quality control and monitoring protocols for milk processing steps leading to cheese manufacture. Evaluating this approach for other cheese types and spoilage defects at other creameries as well as for other food and ingredient types should result in robust methods for rapid detection of spoilage microbes and new opportunities for reducing their contamination and growth of foods.

## MATERIALS AND METHODS

### Milk and cheese sample collection for microbiota assessments.

Milk and cheese samples were collected on 10 days distributed over 4 months in 2015. Milk was obtained from raw milk storage silos, the inlet to the continuous-flow pasteurizer at the final high-temperature, short-time (HTST) pasteurization step (pre-HTST) (71.7°C [161°F]), the outlet from that pasteurizer (post-HTST pasteurization), and cheese blocks made with the post-HTST-pasteurization milk (Fig. 1). Four storage silos were randomly sampled on 7 July 2015, 15 July 2015, 20 July 2015, 29 July 2015, 18 August 2015, 6 October 2015, and 15 October 2015 at 12:00 (noon), on 25 August 2015 at 14:00, and on 29 September 2015 and 19 October 2015 at 11:00. On those same dates, seven sets of pre- and post-HTST-pasteurization milk samples were collected after cleaning-in-place (CIP) protocols were completed for the HTST pasteurizer. The sets were collected after CIP at approximately 0 h (collected at 8:00 and 20:00), 3 h (collected at 11:00 and 23:00), 6 h (collected at 14:00 and 2:00), and 9 h (17:00) on all dates except for 29 July 2015. On 29 July 2015, milk was collected at only 8:00, 11:00, 14:00, and 17:00. Because the length of time needed for milk to pass through the pasteurizer was known, the pre- and post-HTST pasteurization samples were paired such that the milk entering the pasteurizer was collected as it exited after HTST treatment. Raw milk from the storage silos was not paired with the pre- and post-HTST pasteurization samples. Cheddar cheese blocks included in the analysis were made on the same dates between 15:00 and 16:00, corresponding to pre- and post-HTST-pasteurization milk collected at 11:00. The cheese was manufactured using commercial *Lactococcus* starter cultures at 10^9^ CFU/g (DSM, Parsippany, NJ) and formed into 18-kg (40-lb) blocks. Cheese was sampled immediately after it was made (0 days) and after aging at 4°C for 30, 90, and 120 days. For aging, the cheese blocks were stored in vacuum-sealed bags at 4°C. Cheese produced on 15 July 2015, 18 August 2015, 29 September 2015, and 6 October 2015 developed slits within 90 days of aging.

All samples were shipped to the Marco lab (University of California, Davis) overnight on ice and were processed immediately upon arrival. We previously determined that transport in that manner did not significantly affect bacterial composition (4, 17). Milk samples were processed as previously described (4). For cheese samples, 500-g slices were first cut by from the 18-kg blocks using a sterile knife and shipped to the Marco lab. Next, at least five random segments were cut from multiple areas of the slices, including inner (core) outer portions but excluding 1 in. from the edge, to reach a combined total of 30 g. The segments were then placed in Whirl-Pak filter bags (Nasco, GA, USA) with 270 ml 2% (wt/vol) Na_3_C_6_H_5_O_7_ solution and homogenized using a stomacher (Weber Scientific, NJ, USA) at high speed for 10 min. Bacterial cells were separated from 25 ml milk or the cheese-Na_3_C_6_H_5_O_7_ homogenate by centrifuging at 13,000 × *g* for 5 min at 4°C. The cell pellets were washed with phosphate-buffered saline (PBS), pH 7.4 (4). The cell pellets were then either directly frozen at −20°C or were first incubated with propidium monoazide (PMA; Biotium, CA, USA) as previously described (17). Briefly, for PMA treatment, the cells were suspended in PBS containing 50 μM PMA and incubated for 5 min at 23°C. Subsequently, the suspensions were exposed to a 500-W halogen light bulb held 20 cm away for another 5 min with samples inverted every minute and then collected by centrifugation, washed in PBS, and frozen at −20°C.

### Genomic DNA extraction, PCR amplification, and DNA sequencing.

Genomic DNA was extracted from the frozen cell pellets using the MagMAX total nucleic acid isolation kit (Thermo Fisher Scientific, MA, USA) according to the manufacturer’s protocol with the repeat bead-beating method at 6.5 m/s twice for 1 min each time with a 1-min interval on ice. PCR was performed as previously described with Ex Taq polymerase (TaKaRa, Otsu, Japan) and the primers F515 (GTGCCAGCMGCCGCGGTAA) and R806 (GGACTACHVGGGTWTCTAAT), targeting the 16S rRNA gene V4 region, with 8-bp random barcoded sequences on the 5′ end of the forward primer (17). For each DNA extraction kit used (six boxes in total), sham DNA extractions (negative controls) were performed in triplicate for the subsequent PCR amplification and DNA sequencing steps. Equal quantities of PCR products were pooled and then gel purified with the Wizard SV gel and PCR clean-up system (Promega, WI, USA).

Pooled and purified 16S rRNA gene V4 products were sequenced in five separate runs with the Ion Torrent PGM sequencer as previously described (43). Milk and cheese samples from different collection locations, dates, and time points were randomly selected for each run to minimize batch effects. Additionally, in each run, a PCR amplicon mock community (43) was included in triplicate in order to examine for batch effects.

### 16S rRNA gene sequence analysis.

Ion Torrent output BAM files were converted to FASTQ files using BEDTools (44). Reads shorter than 200 bases were discarded. Barcode sequences were extracted by the QIIME 1.9.1 (45) extract_barcodes.py script with no barcode error allowed and were subsequently analyzed in QIIME 2 version 2018.4 (46). Specifically, sequence files were demultiplexed using the demux plugin with the emp-single option. Feature table construction and chimera removal were performed for each run using the DADA2 method (47) and the denoise-single option with default settings, except that the first 29 bases and low-quality bases after position 260 were truncated. Batch effects between the five sequencing runs were determined to be not significant according to the gPCA R package (data not shown) (48). Therefore, feature tables and sequences were merged with the feature-table merge and feature-table merge-seqs plugins, followed by *de novo* read alignment with MAFFT (49). Unconserved and gapped alignments were filtered by the alignment mask plugin with default parameters. A phylogenetic tree was created with FastTree (50) using the filtered alignment method. For taxonomy assignment, a custom classifier was trained based on the truncated sequence reads (231 bases) against the Greengenes database version 13.8 (51).

The merged feature table, feature sequences, rooted phylogenetic tree, and sample information were imported to phyloseq 1.22.3 (52) in R 3.4.2 (53) and visualized with ggplot2 (54) and Superheat (55) packages. *Archaea* (0.04% of total sequence reads), mitochondrion (0.0003% of total sequence reads), and chloroplast (0.4% of total sequence reads) ASVs as well as ASVs that were unidentified at the phylum level (0.65% of total sequence reads) were removed. ASVs making up less than 0.005% of reads from all samples were also removed. For alpha and beta diversity analysis, DNA sequences were rarefied to a depth of 4,000 reads per sample. Significant differences in alpha diversity and sample clustering were determined as previously described (43).

### Bacterial enumeration and identification with qPCR.

Bacterial cell numbers in pre- and post-HTST-pasteurization milk were estimated by qPCR using the primers UniF (GTGSTGCAYGGYYGTCGTCA) and UniR (ACGTCRTCCMCNCCTTCCTC) and compared to the *Lacticaseibacillus* (formerly *Lactobacillus* [56]) *casei* BL23 DNA standard curve as previously described (4). *Turicibacter* sp. numbers were estimated by qPCR targeting the genus-specific 16S rRNA gene region using the primers TuriciF (CAGACGGGGACAACGATTGGA) and TuriciR (TACGCATCGTCGCCTTGGTA) (57) and a standard curve as previously described (17). Lactobacillus helveticus and *Limosilactobacillus* (formerly *Lactobacillus* [56]) *fermentum* numbers were determined by qPCR with species-specific primers (LbhelvF1, AGGTTCAAAGCATCCAATCAATATT; LbhelvR1, TCGGGACCTTGCACTACTTTATAAC; LfermF, GCACCTGATTGATTTTGGTCG; and LfermR, GTCCATTGTGGAAGATTCCC) (58, 59), and standard curves were constructed using DNA isolated from known quantities of L. helveticus NRRL B-4526 and *L. fermentum* NRRL B-1840 cells grown in MRS (Becton, Dickinson and Company, NJ, USA). L. helveticus NRRL B-4526 and *L. fermentum* NRRL B-1840 genomic DNA was extracted following the protocol used for milk and cheese DNA extractions. Reactions were performed with the SsoFast Evagreen Supermix with low ROX (Bio-Rad Laboratories, CA, USA), 400 nM primer mixtures, and 2 μl template DNA. qPCR was performed in duplicate, and reactions yielding a standard deviation of >0.15 were repeated to confirm accuracy.

### Chemical analysis of Cheddar cheese.

Cheddar cheese was sampled after aging at 4°C for 5 days and 90 days. pH and percentages (wt/wt) of salt, moisture, and fat were determined using previously established methods (60). Concentrations (per weight) of galactose, lactose, l-lactate, d-lactate, and citrate were quantified using enzymatic methods with the Gallery Plus discrete photometric system following the manufacturer’s protocol (Thermo Scientific, CA, USA).

### Bacterial isolation and identification.

Milk and cheese sampled on several dates in 2017 (26 April 2017, 6 July 2017, and 25 October 2017) and 2018 (15 February 2018, 22 March 2018, and 23 May 2018) were used for bacterial isolation. Cheeses from 6 July 2017, 25 October 2017, 15 February 2018, and 23 May 2018 developed slits after aging at 4°C for 90 days, whereas those from 26 April 2017 and 22 March 2018 did not. The cheeses were sampled after 5 and 90 days of aging. Samples were collected by randomly cutting the cheese with a sterile knife and collecting 30 g for homogenization in 270 ml 2% (wt/vol) Na_3_C_6_H_5_O_7_ as described above. To select for LAB, serial dilutions of the milk were plated onto MRS agar and incubated for 48 h at 30°C under anaerobic conditions in a GasPak jar (Becton, Dickinson and Company, NJ, USA). To enrich for endospore-forming bacteria, milk and the cheese-Na_3_C_6_H_5_O_7_ homogenate samples were exposed to 80°C for 20 min before plating serial dilutions on brain heart infusion (BHI) agar (Thermo Scientific, MA, USA) and incubating for 48 h at 30°C. These conditions were set because they were sufficient to inactivate the high numbers of L. lactis cells (10^9^ cells/ml) found in Cheddar cheese. Bacterial colonies with distinct morphologies were streaked for isolation twice on MRS or BHI agar before preservation at −80°C.

To identify the bacterial isolates, single colonies were transferred to sterile water and lysed in a microwave at the highest setting for 3 min. PCR amplification was performed with Ex Taq DNA polymerase and the primers 27F (AGAGTTTGATCCTGGCTCAG) and 1492R (GGTTACCTTGTTACGACTT) (61). Amplicons were purified on a Wizard SV gel and PCR clean-up system to prepare for DNA sequencing at Genewiz. Species identity was determined using BLASTn (62) based on 100% nucleotide alignment to the NCBI nr/nt database and confirmed using the Ribosomal Database Project (RDP) database (63).

For testing individual isolates in cheese fermentations, rifampin-resistant mutants of *L. fermentum*, Leuconostoc mesenteroides, and Leuconostoc lactis were obtained as previously described (64). These mutants and *Lactiplatibacillus* (*Lactobacillus*) *plantarum* NCIMB8826-R (65) were routinely grown at 30°C in MRS containing 50 μg/ml of rifampin (Thermo Fisher Scientific, MA, USA).

### CO_2_ production.

Gas production was detected using Durham tubes (66). LAB were incubated in MRS broth, and thermoduric and endospore-forming bacterial isolates were incubated in both MRS and BHI broth for 48 h at 30°C before visual inspection for gas bubbles in the Durham tube.

### Heat tolerance.

LAB strains were suspended in ultrahigh-temperature (UHT) pasteurized milk (Gossner Foods, Inc., UT) at levels ranging from 1.3 × 10^5^ to 8.9 × 10^5^ cells/ml. The cell suspensions were then exposed to 72°C for either 15 s, 1 min, or 5 min. Thermal tolerance was determined by plating serial dilutions of the cell suspensions onto MRS agar for incubation at 30°C for 48 h prior to colony enumeration.

### Pilot-scale Cheddar cheese.

Commercially prepared, fresh cheese curds made using Lactococcus lactis (10^9^ CFU/g) starter culture (DSM) and rennet were collected after salting and draining. One batch of cheese curds was used to make pilot-scale cheeses inoculated with milk consortia and the bacterial strains inoculated at a level of 10^9^ CFU/g. Another batch of curds produced in the same facility was used to make pilot-scale cheeses inoculated with individual strains at a level of 10^7^ CFU/g. For testing individual strains, *L. plantarum* NCIMB8826-R (65) and rifampin-resistant mutants of three isolates of *L. fermentum*, *Ln. mesenteroides*, and L. lactis (Table 1) were grown in MRS at 30°C for approximately 16 h. Cells were collected by centrifugation at 13,000 × *g* for 5 min at 4°C, washed twice with PBS, and suspended in sterile physiologic saline (0.85% NaCl). Bacterial numbers were quantified by microscopy and adjusted such that a 5-ml suspension was added to 260 g cheese curds contained in a Whirl-Pak sampling bag (Nasco, GA, USA) to reach a final cell number of either 10^7^ or 10^9^ CFU/g. Cheese curds inoculated with 5 ml saline were used as controls. Cheese curds were thoroughly mixed with the inoculum by manually massaging the Whirl-Pak sampling bag for 5 min.

**TABLE 1:** Strains and cell consortia used for pilot-scale Cheddar cheese production

Strain or consortium	Origin	Collection date^*a*^	Collection time
*L. fermentum* 3494-1LAB-R	Post-HTST	7/6/17	21:36
*L. fermentum* 3500-3LAB-R	Post-HTST	7/7/17	04:20
*L. fermentum* 3854-3LAB-R	Post-HTST	5/23/18	20:20
*Ln. mesenteroides* 3490-3LAB-R	Pre-HTST	7/6/17	12:10
*Ln. mesenteroides* 3502-1LAB-R	Pre-HTST	7/6/17	21:36
*Ln. mesenteroides* 3504-3LAB-R	Pre-HTST	7/7/17	04:20
*Ln. lactis* 3498-2LAB-R	Pre-HTST	7/6/17	21:36
*Ln. lactis* 3850-1LAB-R	Pre-HTST	5/23/18	13:00
*Ln. lactis* 3860-1LAB-R	Pre-HTST	5/24/18	07:00
Milk_No slits	Post-HTST	7/7/15	11:00
Milk_No slits	Post-HTST	7/20/15	11:00
Milk_No slits	Post-HTST	8/25/15	11:00
Milk_Slits	Post-HTST	8/18/15	11:00
Milk_Slits	Post-HTST	9/29/15	11:00
Milk_Slits	Post-HTST	10/6/15	11:00
Milk_Slits	Post-HTST	7/15/15	11:00

a Month/day/year.

For testing cryopreserved bacterial cell pellets from post-HTST-pasteurization milk, four cell pellets from milk associated with cheese slits and three cell pellets associated with cheese that did not contain slits (Table 1) were suspended in 1 ml sterile physiologic saline (0.85% NaCl) and mixed with 260 g cheese curds as described above. Cheese curds inoculated with 1 ml saline were used as controls.

The curds were then transferred to cheese molds (13 cm in height and 9.5 cm in internal diameter) (Winco perforated flatware cylinder; Winco, Lodi, NJ), lined with two layers of cheesecloth, and pressed for 15 min using an A-frame vertical cheese press (Ullmer, Pulaski, WI) at 8 lb/in^2^ to remove extra whey and form the cheese wheel. As performed for the commercial cheese blocks, the cheese wheels were then vacuum packed. The cheese was aged at 30°C to expedite ripening (67–69). The elevated aging temperature is also consistent with expected temperatures near the core of commercial cheese blocks, which can take up to 12 days to completely cool down to 4°C (data not shown) (70). Cheeses inoculated with bacterial cells at a level of 10^9^ CFU/g were incubated for 5 days, and cheeses inoculated with 10^7^ CFU/g or cryopreserved milk cell were incubated for 11 days.

### Bacterial enumeration and slit quantification of pilot-scale cheese.

After aging, pilot cheeses were cut randomly with a sterile knife to collect a total of 3 g cheese from at least five locations of the cheese wheel, including the inner and outer portions. The cheese samples were then homogenized in 27 ml 2% (wt/vol) Na_3_C_6_H_5_O_7_ as described above. Serial dilutions of the cheese homogenates were prepared in PBS prior to plating onto tryptic soy agar (Becton, Dickinson and Company, NJ, USA), MRS agar, and MRS agar containing 50 μg/ml rifampin, for detection of the culturable bacteria, LAB, and rifampin-resistant LAB strains, respectively. Colony enumeration was performed after incubating the plates at 30°C for 48 h. For image quantification, three images of the randomly cut cheese cross sections were analyzed by ImageJ (71). Slit area was calculated as a percentage of the cheese cross-sectional slice as follows: (pixels of slits/pixels of cheese cross section) × 100.

### Statistics.

For the 16S rRNA gene DNA sequencing results, differential taxa abundance between sample groups was determined between unrarefied samples using DESeq2 version 1.18.1 (72) and was considered significant when the *P* value after Benjamini-Hochberg correction was less than 0.1 and fold change was more than 1.25. For taxa with geometric means equal to zero, the geometric mean was replaced by pseudocounts (1) before the DESeq() function.

To determine significance differences between bacterial alpha diversity, qPCR-estimated cell numbers, and slit area, the Mann-Whitney test (*P* < 0.05) was used for pairwise comparison and the Kruskal-Wallis with Dunn test (*P* < 0.05) was used for multiple comparisons. Odds ratio analysis was performed using the R package questionr (73). Power analysis was performed using the R package pwr (74) with 0.05 as the significance level for a single-tailed comparison (“greater”) and various effect sizes, sample sizes, and power values.

### Availability of data.

DNA sequences were deposited in the Qiita database (75) under study ID 12366 (https://qiita.ucsd.edu/study/description/12366#) and in the European Nucleotide Archive (ENA) under accession number ERP114733 (https://www.ebi.ac.uk/ena/data/view/PRJEB32097).
