# Supplementary figures and images for: Microbiota Assessments for the Identification and Confirmation of Slit Defect-Causing Bacteria in Milk and Cheddar Cheese
Source: mSystems. 2021 Feb 9;6(1):e01114-20. doi: 10.1128/mSystems.01114-20 (PMC7883541; doi:10.1128/mSystems.01114-20)

**A**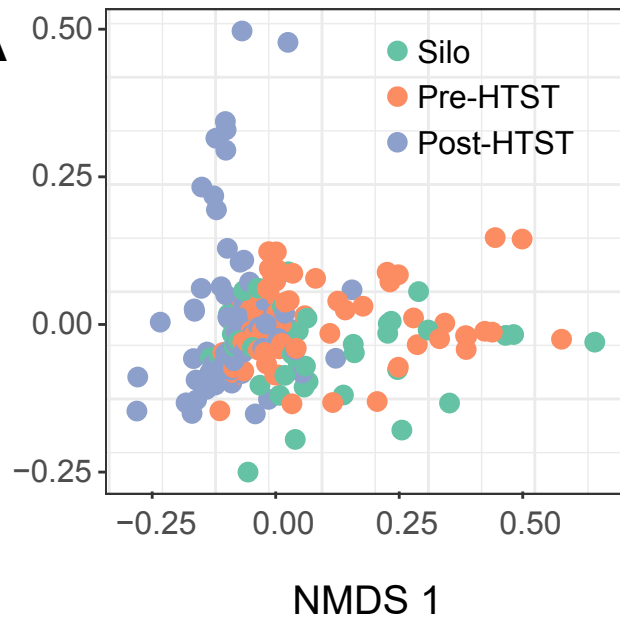**B**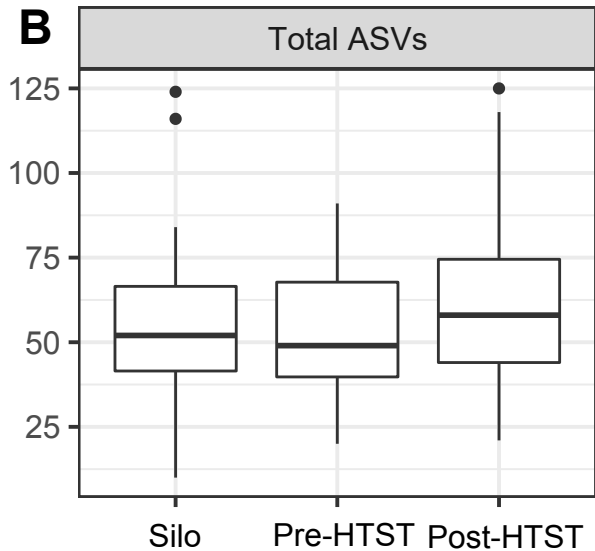**C**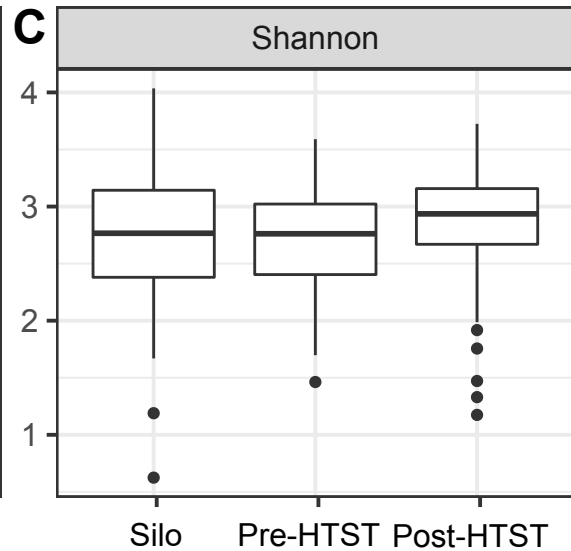

Supplement: FIG S1 [file mSystems.01114-20-sf001.pdf]

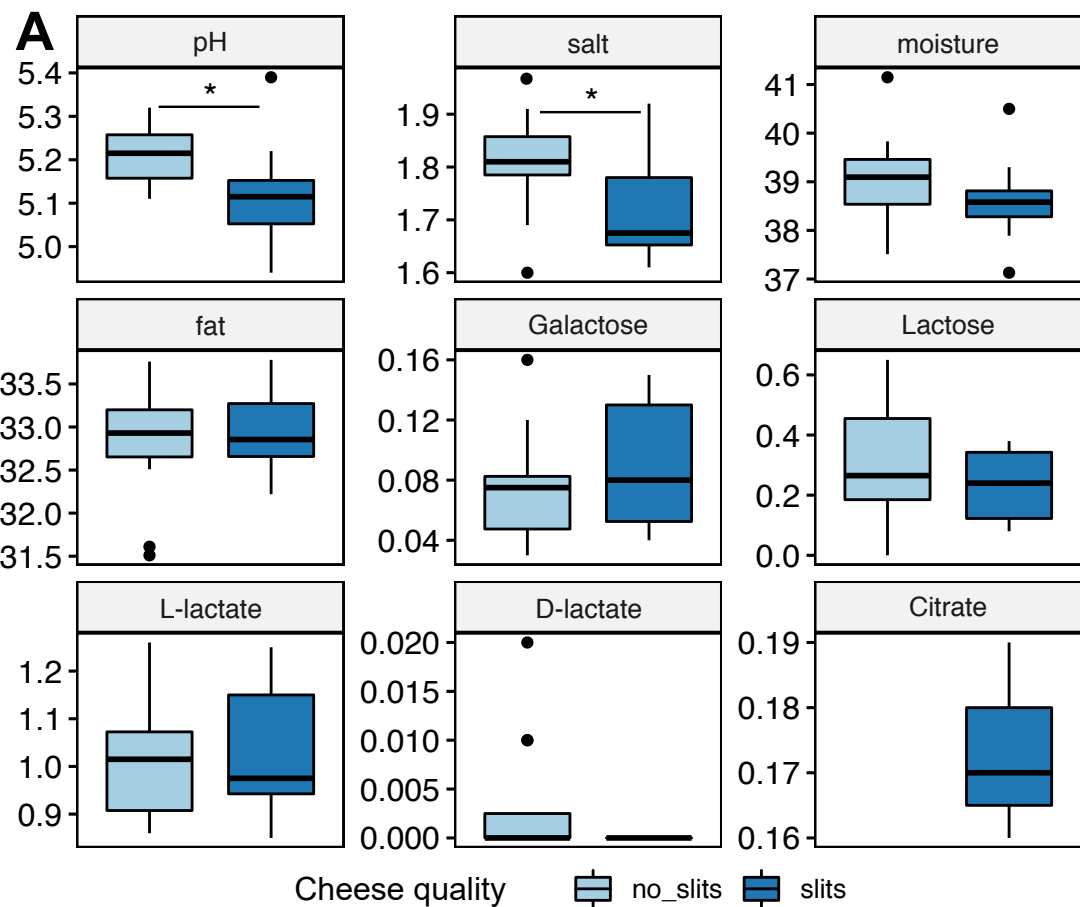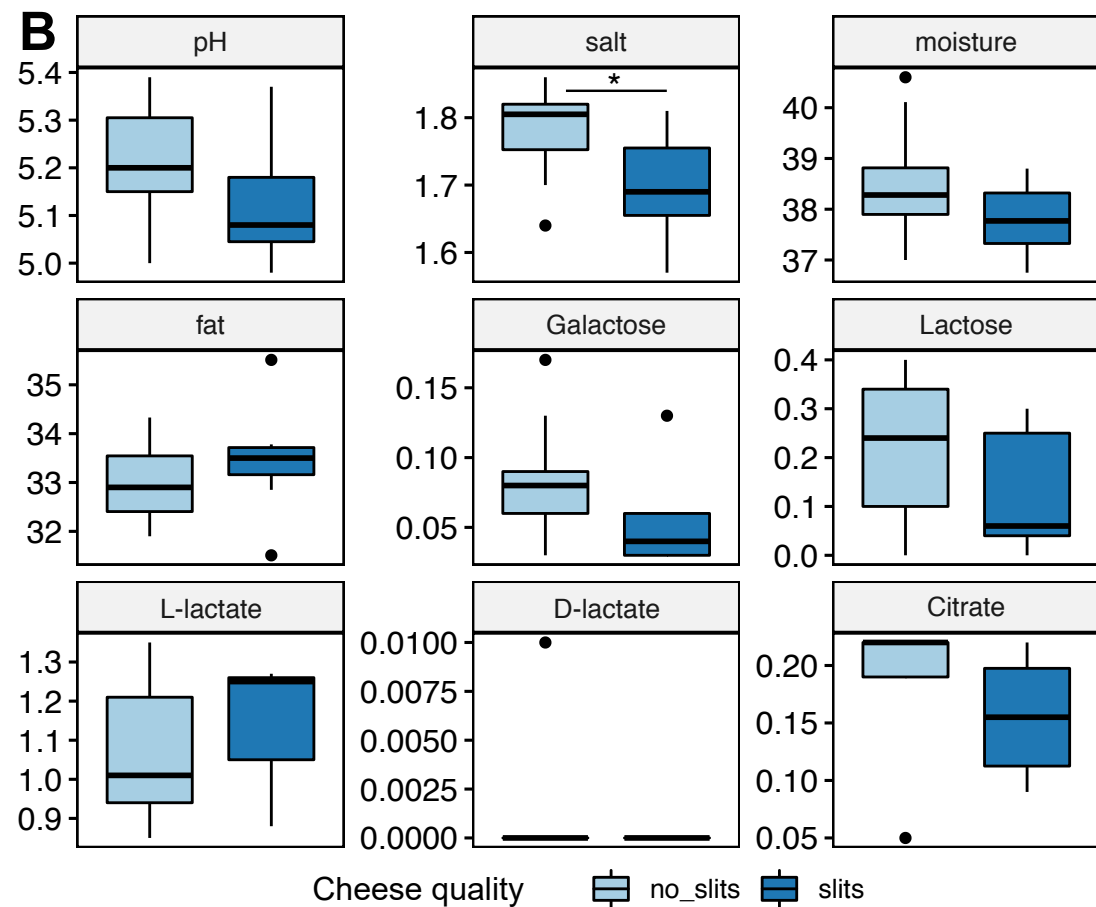

Supplement: FIG S2 [file mSystems.01114-20-sf002.pdf]

*Turicibacter*

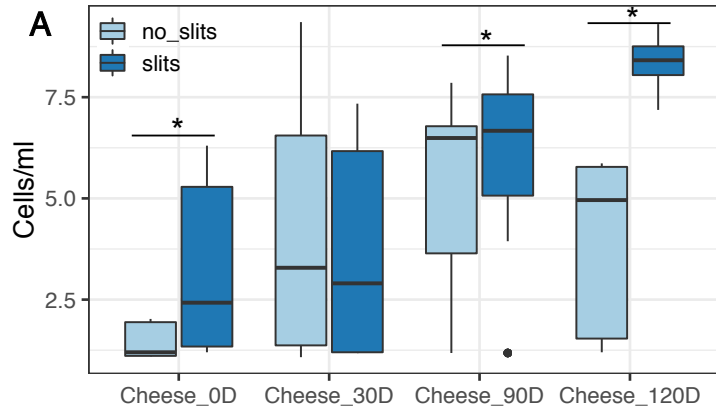

*L. fermentum*

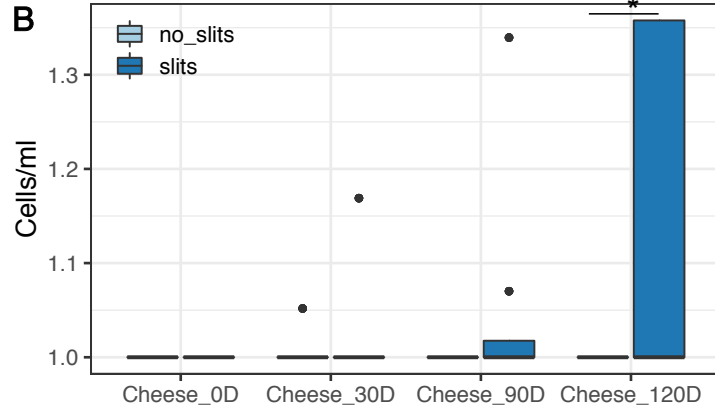

*L. fermentum*

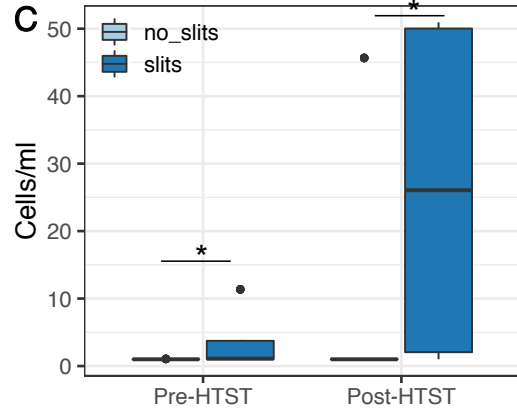

Supplement: FIG S3 [file mSystems.01114-20-sf003.pdf]

Relative abundance

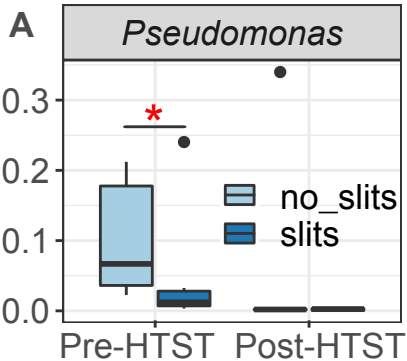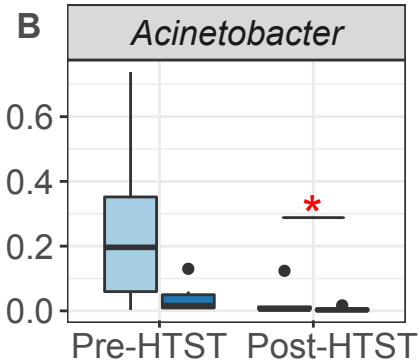

Supplement: FIG S4 [file mSystems.01114-20-sf004.pdf]

Pre-HTST

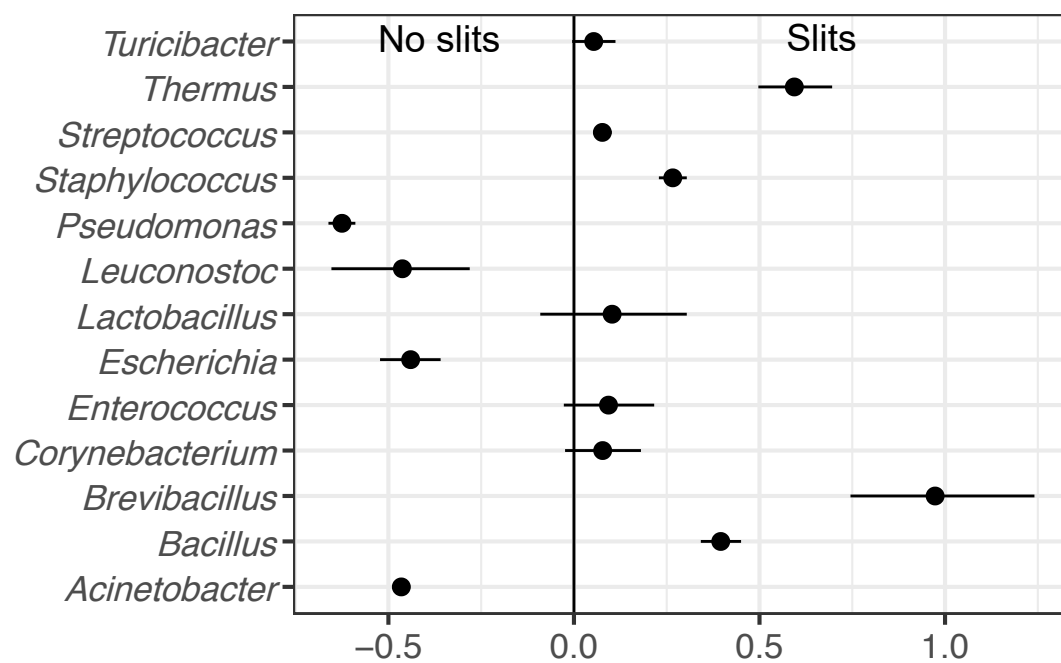

Post-HTST

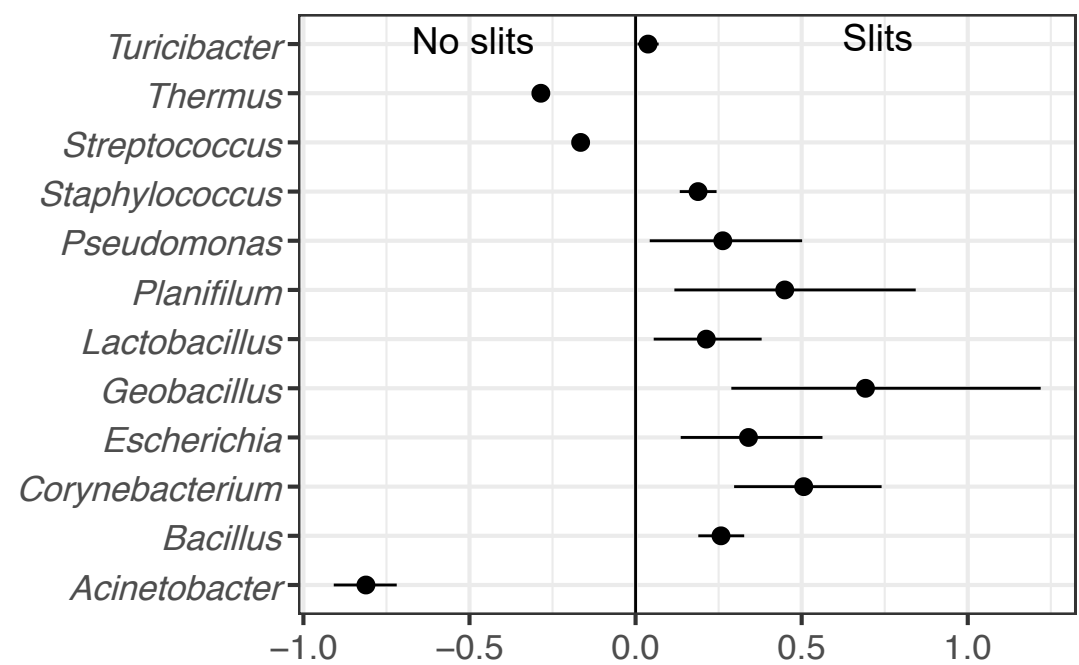

Cheese 0D

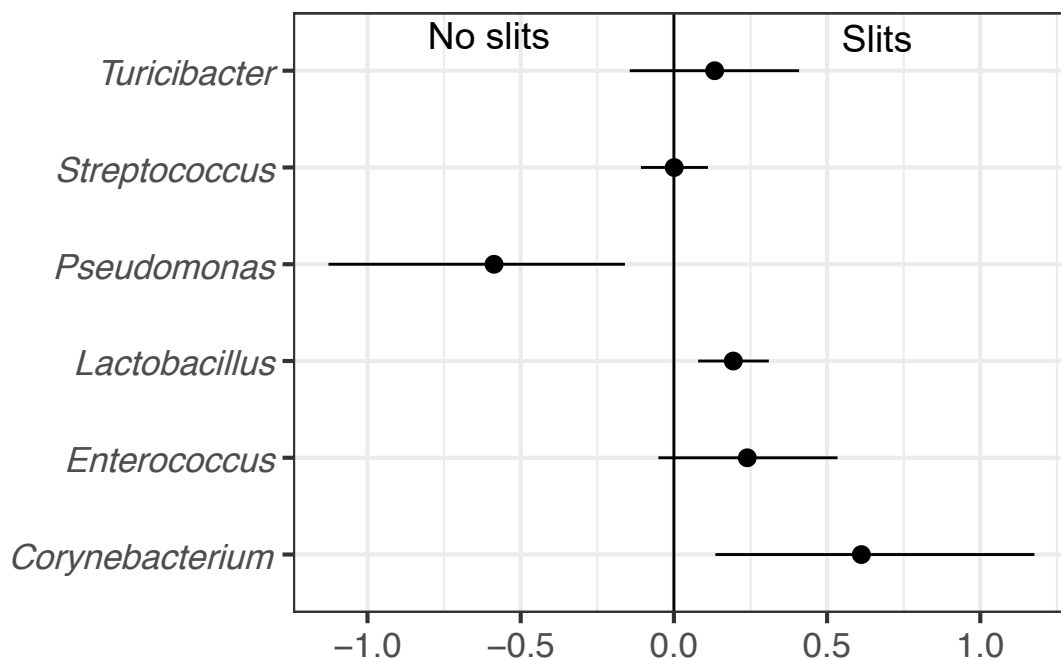

Cheese 30D

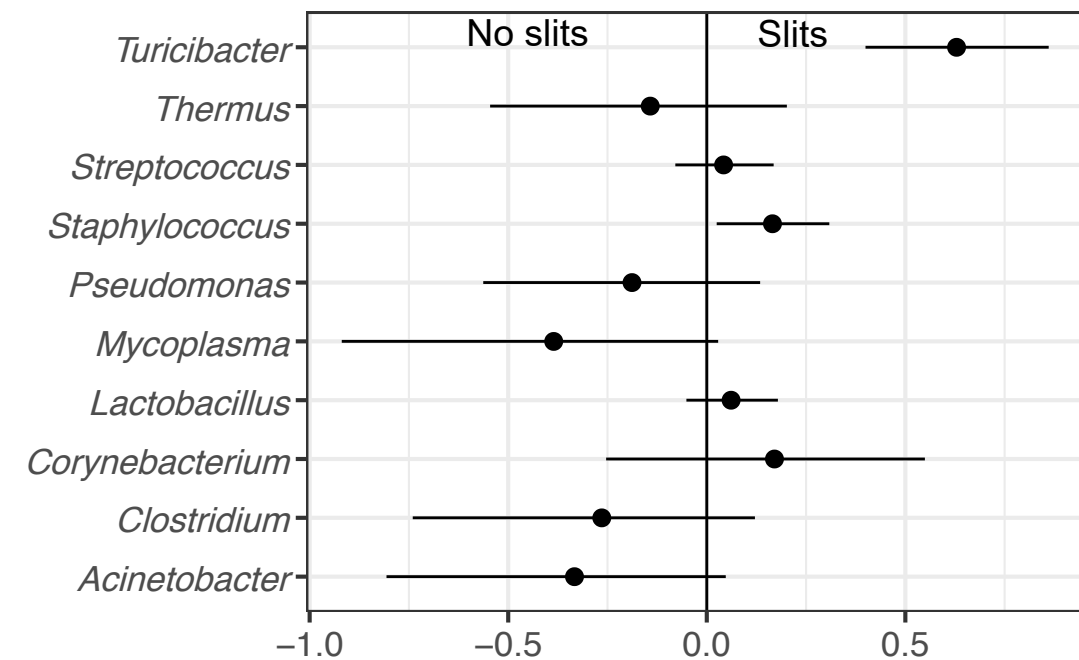

Cheese 90D

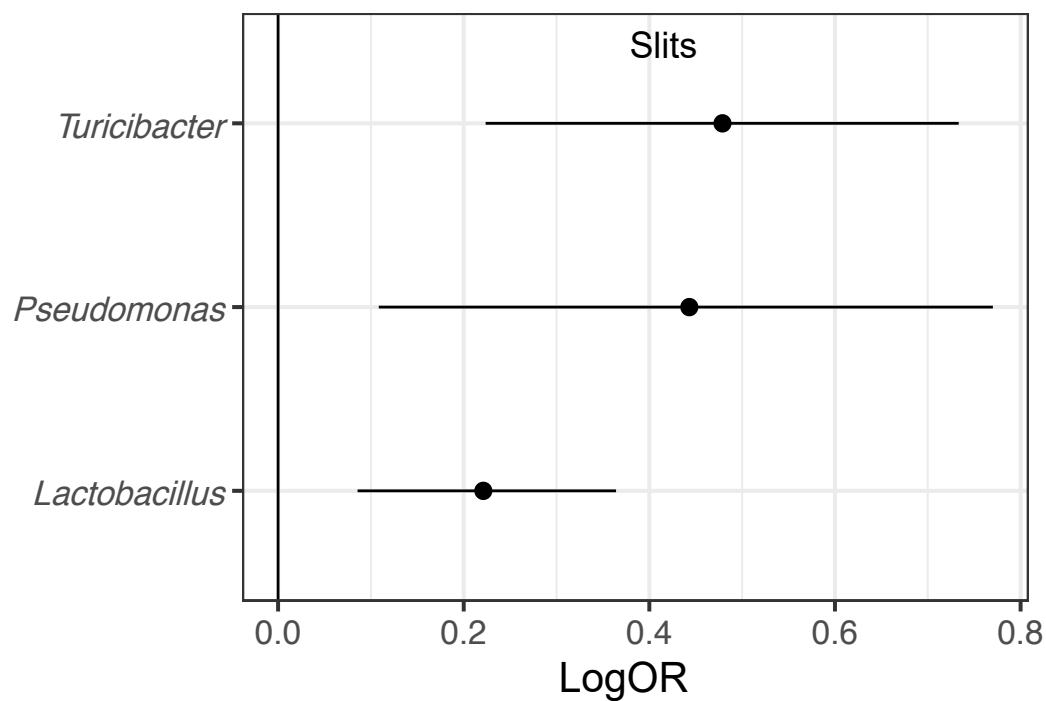

Cheese 120D

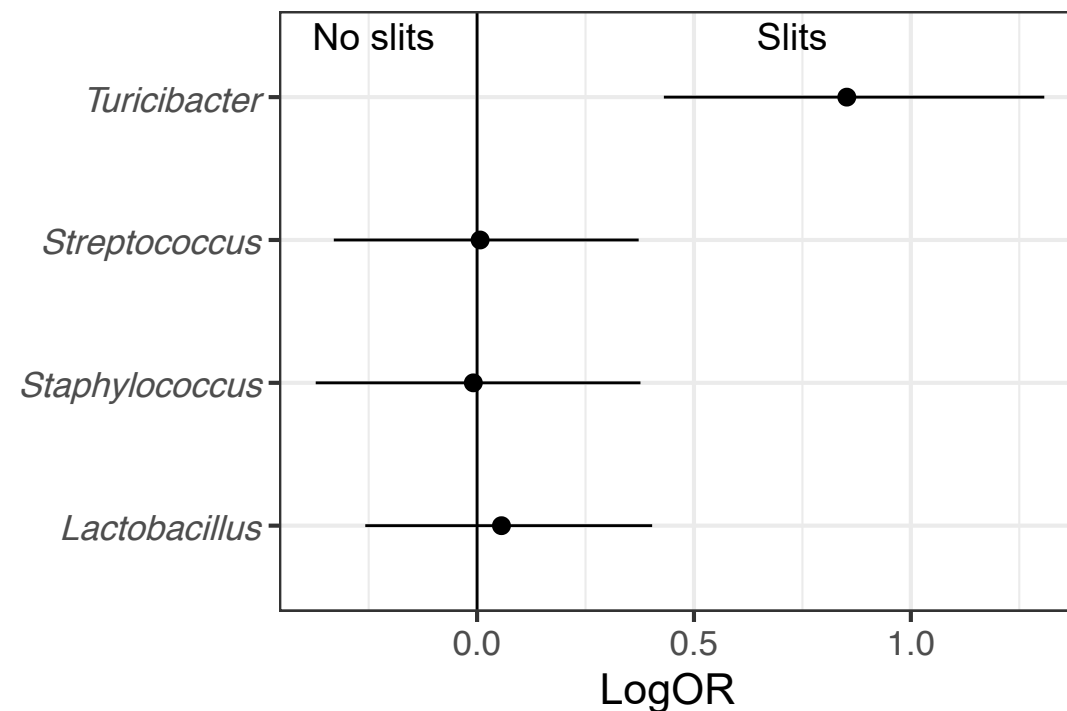

Supplement: FIG S5 [file mSystems.01114-20-sf005.pdf]

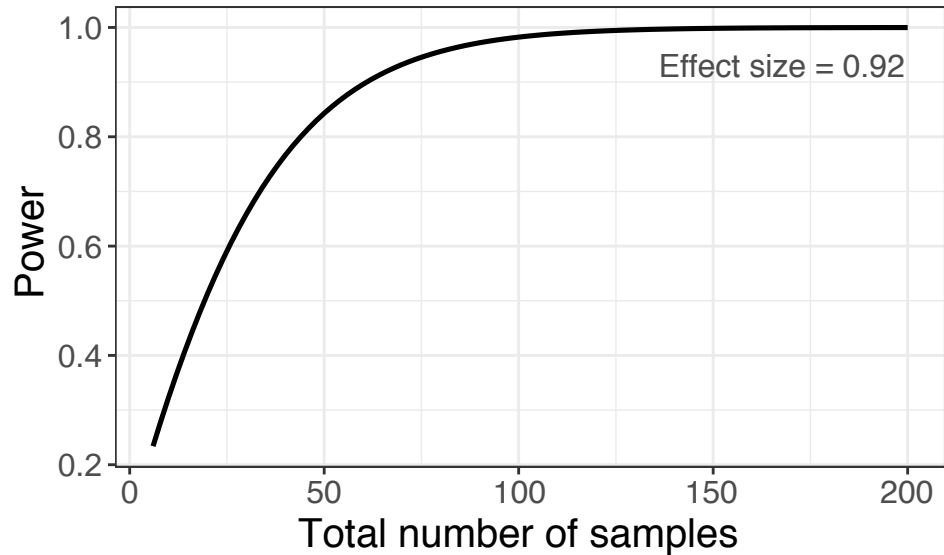

Supplement: FIG S6 [file mSystems.01114-20-sf006.pdf]

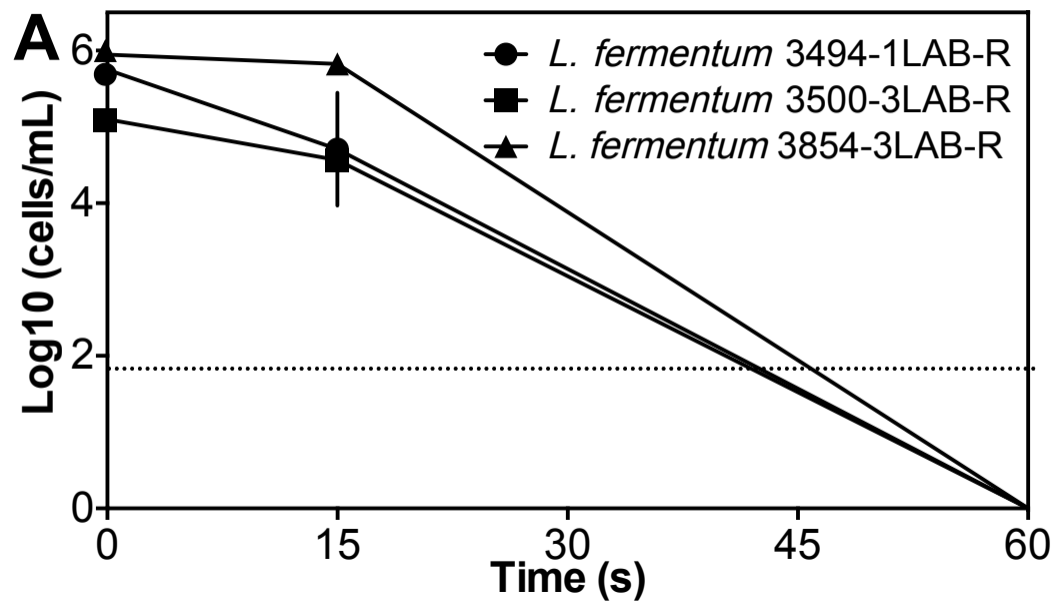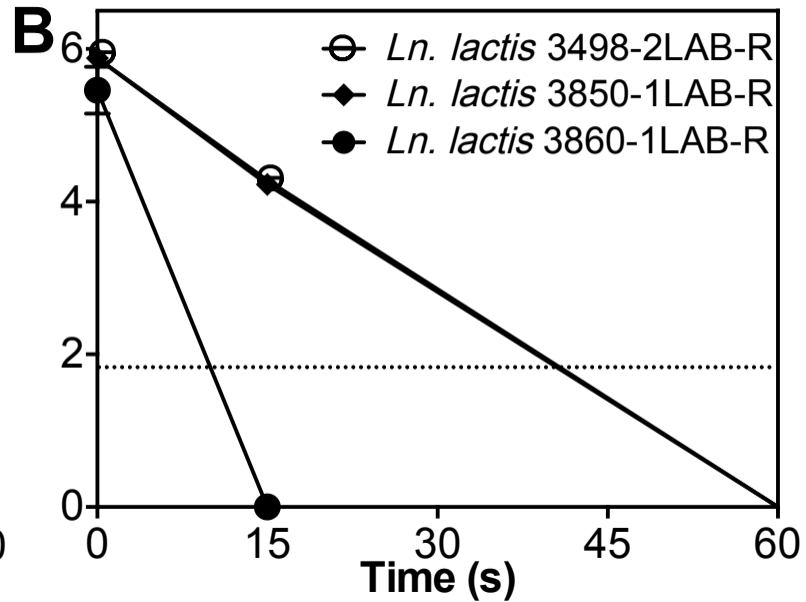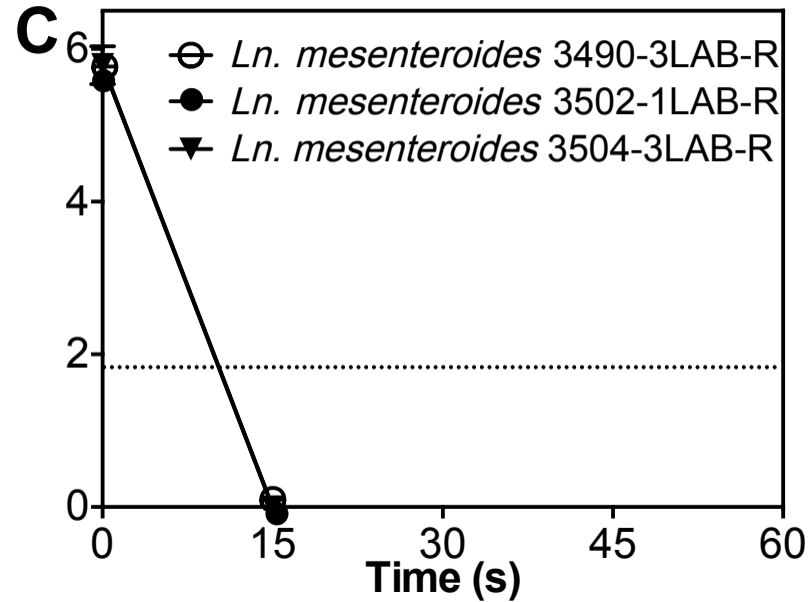

Supplement: FIG S7 [file mSystems.01114-20-sf007.pdf]

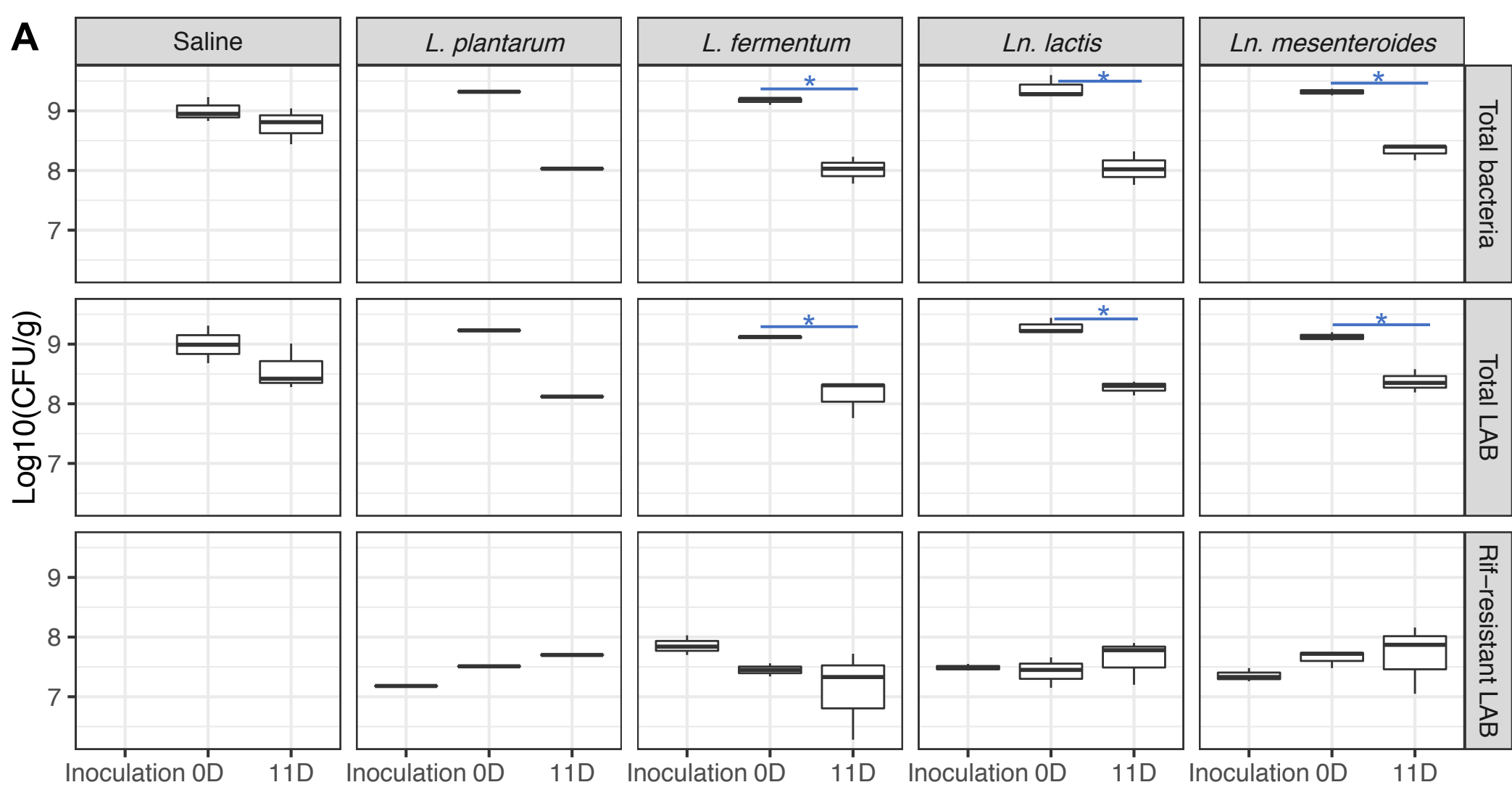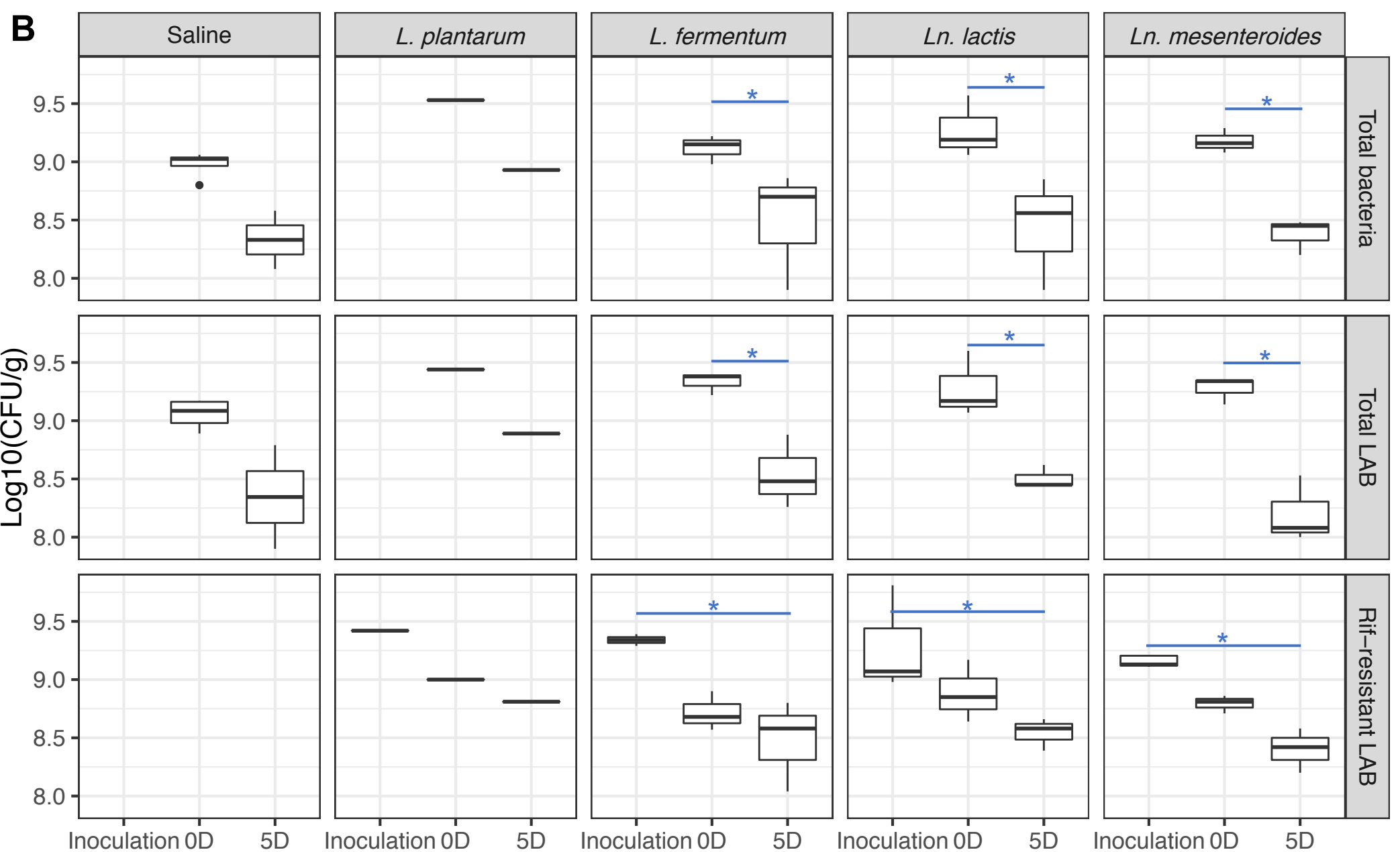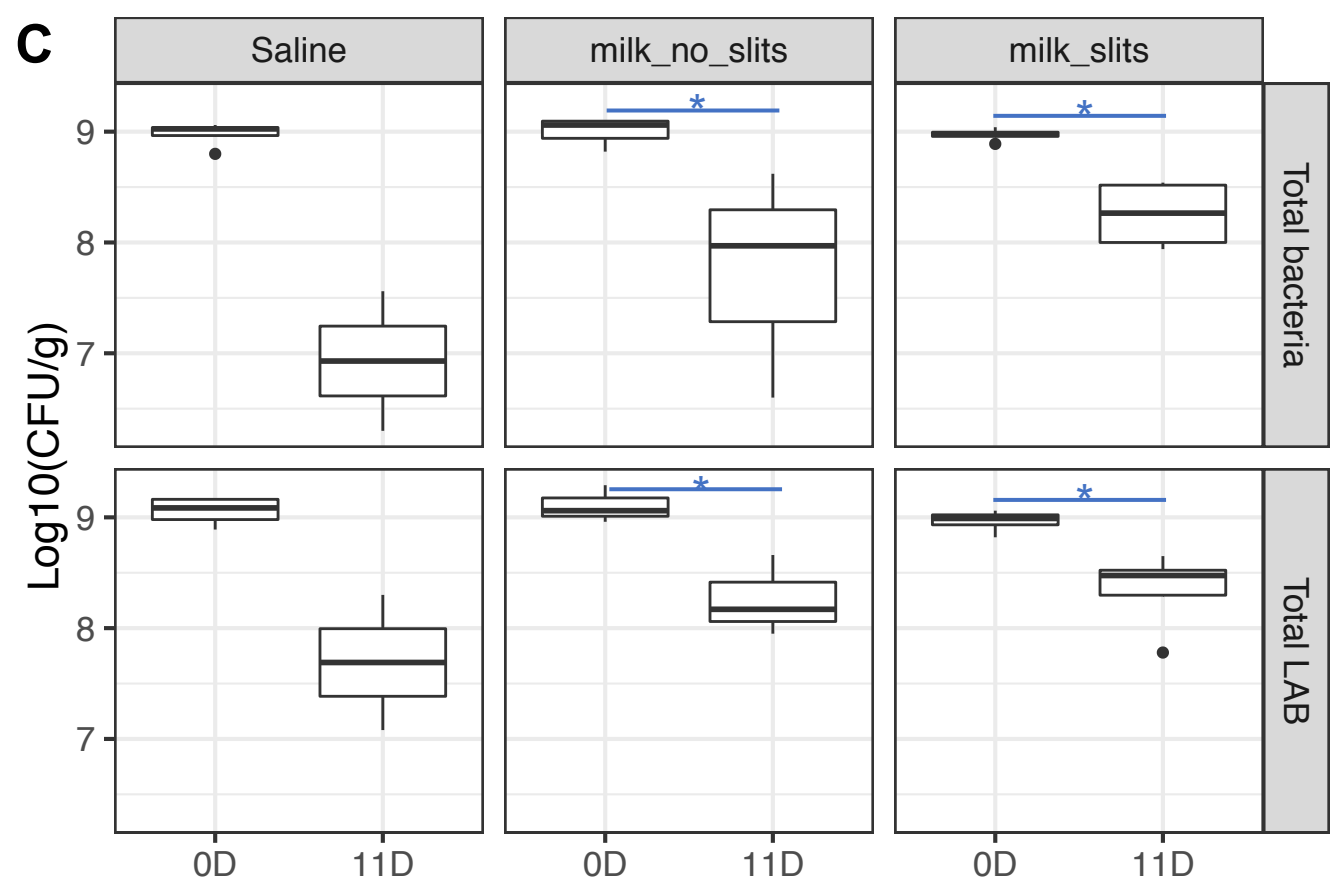

Supplement: FIG S8 [file mSystems.01114-20-sf008.pdf]

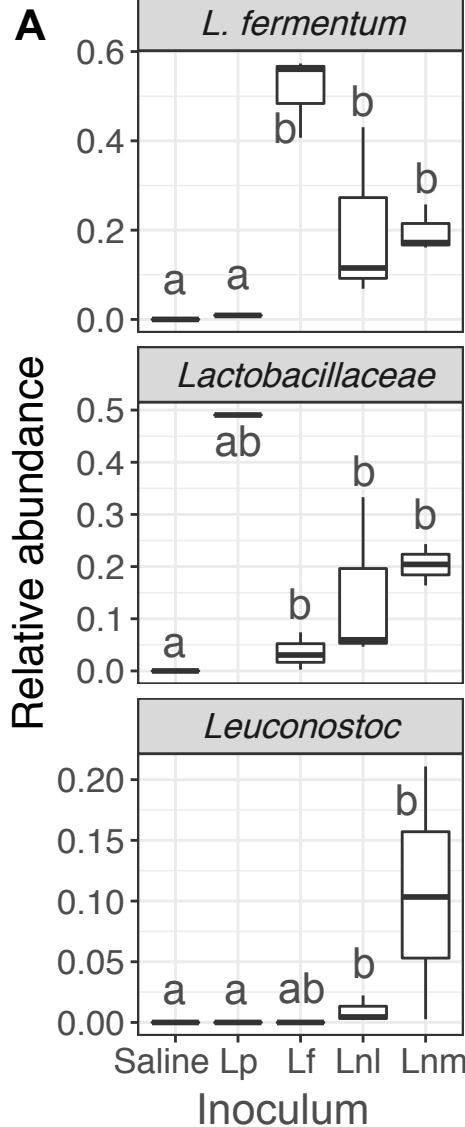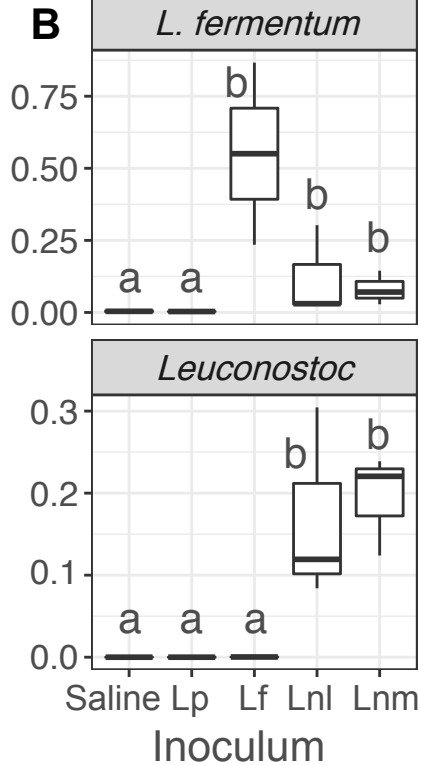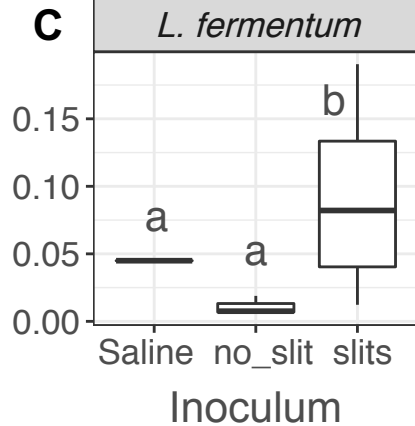

Supplement: FIG S9 [file mSystems.01114-20-sf009.pdf]
