# Supplementary material for: Microbiota Assessments for the Identification and Confirmation of Slit Defect-Causing Bacteria in Milk and Cheddar Cheese
Source: mSystems. 2021 Feb 9;6(1):e01114-20. doi: 10.1128/mSystems.01114-20 (PMC7883541; doi:10.1128/mSystems.01114-20)
Supplement: TABLE S1 [file mSystems.01114-20-st001.docx]

**Table S1. Numbers of LAB and thermoduric bacteria isolated from milk pre-HTST and post-HTST and from Cheddar cheese.**

|  | |  | **Collection date ^a^** | | | | | | | | | | | | | | | |  |
| --- | --- | --- | --- | --- | --- | --- | --- | --- | --- | --- | --- | --- | --- | --- | --- | --- | --- | --- | --- |
|  | |  | **Pre-HTST** | | | | **Post-HTST** | | | | | | **Cheese** | | | | | |  |
|  | |  | **4/26/17** | **7/6/17** | **3/22/18** | **5/23/18** | **4/26/17** | **7/6/17** | **10/25/17** | **2/15/18** | **3/22/18** | **5/23/18** | **4/26/17** | **7/6/17** | **10/25/17** | **2/15/18** | **3/22/18** | **5/23/18** |  |
| **Lactic acid bacteria ^b^** | | ***Lactobacillus delbrueckii subsp. lactis*** | |  |  |  |  |  |  |  |  |  | 1 |  |  |  |  |  |  |
|  |  | ***Limosilactobacillus fermentum*** | |  | 7 |  | 1 |  | 2 |  |  |  | 1 |  |  |  |  |  |  |
|  |  | ***Lacticaseibacillus paracasei*** | |  | 1 |  | 5 |  |  |  |  |  |  |  |  |  |  |  |  |
|  |  | ***Lactiplantibacillus paraplantarum*** | |  | 2 |  |  |  |  |  |  |  |  |  |  |  |  |  |  |
|  |  | ***Lactiplantibacillus plantarum*** | | 8 | 7 |  |  |  | 1 |  |  |  |  |  |  |  |  |  |  |
|  |  | ***Lacticaseibacillus rhamnosus*** | | 3 |  |  |  |  |  |  |  |  |  |  |  |  |  |  |  |
|  |  | ***Leuconostoc lactis*** | | 1 | 1 |  | 4 |  |  |  |  |  |  |  |  |  |  |  |  |
|  |  | ***Leuconostoc mesenteroides*** | | 2 | 3 | 1 |  |  |  |  |  |  |  |  |  |  |  |  |  |
|  |  | ***Leuconostoc sp.*** | |  |  |  | 1 |  |  |  |  |  |  |  |  |  |  |  |  |
|  |  | ***Pediococcus acidilactici*** | |  | 1 |  |  |  |  |  |  |  |  |  |  |  |  |  |  |
|  |  | ***Streptococcus thermophilus*** | |  |  |  |  |  |  | 5 |  |  |  |  | 3 |  |  | 7 |  |
|  |  | ***Weissella paramesenteroides*** | | 1 |  |  |  |  |  |  |  |  |  |  |  |  |  |  |  |
| ***Bacillales* ^c^** | | ***Bacillus aerius*** | |  |  |  |  |  |  | 2 |  |  |  |  |  |  |  |  |  |
|  |  | ***Bacillus amyloliquefaciens*** | |  |  | 1 |  |  |  |  |  |  |  |  | 1 |  |  |  |  |
|  |  | ***Bacillus circulans*** | |  |  |  |  | 1 |  | 2 |  |  |  |  |  | 2 |  |  |  |
|  |  | ***Bacillus clausii*** | |  |  | 1 |  |  |  | 2 |  |  |  |  |  |  |  | 2 |  |
|  |  | ***Bacillus coagulans*** | |  |  |  |  |  |  |  | 1 |  |  |  |  |  |  |  |  |
|  |  | ***Bacillus firmus*** | |  |  | 2 |  |  |  | 3 |  | 2 |  |  | 2 |  | 5 | 7 | 1 |
|  |  | ***Bacillus flexus*** | |  |  |  |  |  |  | 1 |  |  |  |  |  |  | 1 |  |  |
|  |  | ***Bacillus fordii*** | |  |  |  |  |  |  |  |  |  |  |  | 1 |  |  |  |  |
|  |  | ***Bacillus isronensis*** | |  |  |  |  |  |  |  |  |  |  |  | 1 |  |  |  |  |
|  |  | ***Bacillus kochii*** | |  |  |  |  |  |  | 2 |  |  |  |  |  |  |  |  |  |
|  |  | ***Bacillus licheniformis*** | |  |  | 1 |  | 2 |  | 16 |  |  |  |  | 15 | 6 |  |  | 1 |
|  |  | ***Bacillus megaterium*** | |  |  | 1 |  | 1 |  | 2 |  |  |  |  | 1 |  | 1 |  |  |
|  |  | ***Bacillus niacini*** | |  |  |  |  |  |  | 2 |  |  |  |  |  |  |  |  |  |
|  |  | ***Bacillus novalis*** | |  |  |  |  |  |  |  | 1 |  |  |  |  |  |  |  |  |
|  |  | ***Bacillus oceanisediminis*** | |  |  |  |  |  |  | 9 |  |  |  |  | 3 | 2 |  | 1 |  |
|  |  | ***Bacillus oleronius*** | |  |  |  |  |  |  |  |  | 1 |  |  | 1 |  |  | 1 |  |
|  |  | ***Bacillus paralicheniformis*** | |  |  | 3 |  |  |  |  | 2 | 5 |  |  |  | 8 | 4 | 3 | 5 |
|  |  | ***Bacillus proteolyticus*** | |  |  |  |  |  |  |  |  |  |  |  |  |  |  |  | 1 |
|  |  | ***Bacillus pumilus*** | |  |  |  |  |  |  |  |  | 1 |  |  |  | 3 |  | 1 |  |
|  |  | ***Bacillus rhizosphaerae*** | |  |  |  |  |  |  |  |  |  |  |  | 1 |  |  |  |  |
|  |  | ***Bacillus ruris*** | |  |  |  |  |  |  |  |  |  |  |  | 1 |  |  |  |  |
|  |  | ***Bacillus siralis*** | |  |  |  |  |  |  | 1 |  |  |  |  | 1 |  |  |  |  |
|  |  | ***Bacillus sp.*** | |  |  |  |  |  |  |  |  |  |  | 1 |  |  |  | 1 | 2 |
|  |  | ***Bacillus subtilis*** | |  |  |  |  | 1 |  | 3 |  |  |  |  | 2 |  |  |  |  |
|  |  | ***Bacillus tequilensis*** | |  |  |  |  |  |  |  |  |  |  |  |  |  |  |  |  |
|  |  | ***Bacillus velezensis*** | |  |  | 1 |  |  |  |  | 1 | 2 |  | 1 |  | 1 |  |  |  |
|  |  | ***Bacillus zhangzhouensis*** | |  |  |  |  |  |  | 1 |  |  |  |  | 1 | 1 |  |  |  |
|  |  | ***Brevibacillus brevis*** | |  |  |  |  |  |  |  | 2 |  |  |  |  | 2 |  |  |  |
|  |  | ***Fictibacillus phosphorivorans*** | |  |  |  |  |  |  | 2 |  |  |  |  | 3 |  |  |  | 1 |
|  |  | ***Oceanobacillus sp.*** | |  |  |  |  |  |  |  |  |  |  |  | 3 |  |  |  | 1 |
|  |  | ***Ornithinibacillus scapharcae*** | |  |  |  |  |  |  |  |  |  |  |  | 2 |  |  |  |  |
|  |  | ***Paenibacillus sp.*** | |  |  |  |  | 1 |  |  |  |  |  |  | 1 |  | 2 | 1 | 1 |
|  |  | ***Paucisalibacillus globulus*** | |  |  |  |  |  |  |  |  |  |  | 1 |  |  |  | 1 |  |
|  |  | ***Sporosarcina soli*** | |  |  |  |  |  |  |  |  |  |  |  | 1 |  |  |  |  |
| **Other bacteria ^c^** | | ***Janibacter anophelis*** | |  |  |  |  |  |  |  |  |  |  |  |  |  | 1 |  |  |
|  |  | ***Kocuria varians*** | |  |  |  |  |  |  | 1 |  |  |  |  |  |  |  |  |  |
|  |  | ***Microbacterium lacticum*** | |  |  |  |  |  |  | 6 | 1 |  |  |  |  |  |  |  | 1 |
|  |  | ***Micrococcus luteus*** | |  |  |  |  |  |  |  |  |  |  |  | 1 |  |  |  |  |
|  |  | ***Pseudomonas fragi*** | |  |  | 3 |  |  |  |  |  |  |  |  |  |  |  |  |  |
|  |  | ***Pseudomonas psychrophila*** | |  |  | 2 |  |  |  |  |  |  |  |  |  |  |  |  |  |
|  |  | ***Serratia liquefaciens*** | |  |  | 2 |  |  |  |  |  |  |  |  |  |  |  |  |  |
|  |  | ***Staphylococcus hominis*** | |  |  |  |  |  |  |  |  |  |  |  | 1 |  |  |  |  |

^a^ Cheddar cheese made on 7/6/2017, 10/25/2017, 2/15/2018 and 5/23/2018 developed slits and those dates are shaded gray.

^b^ Because of the high numbers of the LAB *L. lactis* in the cheese, LAB were only isolated from milk samples. *Streptococcus* *thermophilus* were isolated from cheese under thermoduric bacterial enrichment conditions.

^c^ Identified as thermoduric due to survival upon exposure to 80 °C for 20 min.
